# The effect of twice-weekly versus once-weekly sessions of either imagery rescripting or eye movement desensitization and reprocessing for adults with PTSD from childhood trauma (IREM-Freq): a study protocol for an international randomized clinical trial

**DOI:** 10.1186/s13063-021-05712-9

**Published:** 2021-11-27

**Authors:** Carlijn J. M. Wibbelink, Christopher W. Lee, Nathan Bachrach, Sarah K. Dominguez, Thomas Ehring, Saskia M. van Es, Eva Fassbinder, Sandra Köhne, Magda Mascini, Marie-Louise Meewisse, Simone Menninga, Nexhmedin Morina, Sophie A. Rameckers, Kathleen Thomaes, Carla J. Walton, Ingrid G. Wigard, Arnoud Arntz

**Affiliations:** 1 Department of Clinical Psychology, University of Amsterdam, Nieuwe Achtergracht 129-B, 1018 WS Amsterdam, the Netherlands; 2 Faculty of Health and Medical Sciences, University of Western Australia, 35 Stirling Highway, Crawley, Western Australia 6009 Australia; 3 GGZ Oost Brabant, RINO Zuid and Tilburg University, Kluisstraat 2, 5427 EM, Boekel, the Netherlands; 4 School of Psychology and Exercise Science, Murdoch University and Sexual Assault Resource Centre, 90 South Street, Murdoch, Western Australia 6150 Australia; 5 Department of Psychology, LMU Munich, Leopoldstr. 13, 80802 Munich, Germany; 6 PsyQ Amsterdam, Parnassia Groep, Overschiestraat 57, 1062 HN Amsterdam, the Netherlands; 7 Department of Psychiatry and Psychotherapy, Christian-Albrechts-University Kiel, Niemannsweg 147, 24105 Kiel, Germany; 8 Department of Psychiatry and Psychotherapy, University of Lübeck, Ratzeburger Allee 160, 23538 Lübeck, Germany; 9 GGZ Noord-Holland-Noord, Stationsplein 138, 1703 WC Heerhugowaard, the Netherlands; 10 Abate, Centre of Expertise in Anxiety and Trauma, Postweg 3, 1601 SX Enkhuizen, the Netherlands; 11 PsyQ Beverwijk, Parnassia Groep, Leeghwaterweg 1a, 1951 NA, Velsen-Noord, the Netherlands; 12 Institute of Psychology, University of Münster, Fliednerstr. 21, 48149 Muenster, Germany; 13 Sinai Center, the Psychotrauma Expertise Center of Arkin and Amsterdam UMC, location VUmc, Department Psychiatry and Department of Anatomy and Neuroscience, Laan van de Helende Meesters 2, 1186 AM Amstelveen, the Netherlands; 14 Centre for Psychotherapy, Hunter New England Mental Health Service, NSW, Australia, PO Box 833, Newcastle, NSW 2300 Australia

**Keywords:** Posttraumatic stress disorder, Childhood, Imagery rescripting, Eye movement desensitization and reprocessing, Treatment, Randomized clinical trial, Session frequency

## Abstract

**Background:**

Trauma-focused treatments for posttraumatic stress disorder (PTSD) are commonly delivered either once or twice a week. Initial evidence suggests that session frequency affects treatment response, but very few trials have investigated the effect of session frequency. The present study’s aim is to compare treatment outcomes of twice-weekly versus once-weekly sessions of two treatments for PTSD related to childhood trauma, imagery rescripting (ImRs) and eye movement desensitization and reprocessing (EMDR). We hypothesize that both treatments will be more effective when delivered twice than once a week. *How* session frequency impacts treatment response, whether treatment type moderates the frequency effect, and which treatment type and frequency works best for whom will also be investigated.

**Methods:**

The IREM-Freq trial is an international multicenter randomized clinical trial conducted in mental healthcare centers across Australia, Germany, and the Netherlands. We aim to recruit 220 participants, who will be randomized to one of four conditions: (1) EMDR once a week, (2) EMDR twice a week, (3) ImRs once a week, or (4) ImRs twice a week. Treatment consists of 12 sessions. Data are collected at baseline until one-year follow-up. The primary outcome measure is clinician-rated PTSD symptom severity. Secondary outcome measures include self-reported PTSD symptom severity, complex PTSD symptoms, trauma-related cognitions and emotions, depressive symptoms, dissociation, quality of life, and functioning. Process measures include memory, learning, therapeutic alliance, motivation, reluctance, and avoidance. Additional investigations will focus on predictors of treatment outcome and PTSD severity, change mechanisms of EMDR and ImRs, the role of emotions, cognitions, and memory, the optimization of treatment selection, learned helplessness, perspectives of patients and therapists, the network structure of PTSD symptoms, and sudden treatment gains.

**Discussion:**

This study will extend our knowledge on trauma-focused treatments for PTSD related to childhood trauma and, more specifically, the importance of session frequency. More insight into the optimal session frequency could lead to improved treatment outcomes and less dropout, and in turn, to a reduction of healthcare costs. Moreover, the additional investigations will broaden our understanding of how the treatments work and variables that affect treatment outcome.

**Trial registration:**

Netherlands Trial Register NL6965, registered 25/04/2018.

**Supplementary Information:**

The online version contains supplementary material available at 10.1186/s13063-021-05712-9.

## Administrative information

**Table:** 

Title	The Effect of Twice-Weekly Versus Once-Weekly Sessions of Either Imagery Rescripting or Eye Movement Desensitization and Reprocessing for Adults with PTSD from Childhood Trauma (IREM-Freq): A Study Protocol for an International Randomized Clinical Trial
Trial registration	Netherlands Trial Register, NL6965, registered 25/04/2018, https://www.trialregister.nl/trial/6965.
Protocol version	Protocol version 2.1 is currently active.
Funding	This study is partially funded by the EMDR research foundation to pay for a research assistant at one of the Australian sites.
Author details	C.J.M. Wibbelink: University of Amsterdam, the Netherlands C.W. Lee: University of Western Australia, Australia N. Bachrach: GGZ Oost Brabant, RINO Zuid and Tilburg University, the Netherlands S.K. Dominguez: School of Psychology and Exercise Science, Murdoch University and Sexual Assault Resource Centre*,* Australia T. Ehring: LMU Munich, Germany S.M. van Es: PsyQ Amsterdam, Parnassia Groep, the Netherlands E. Fassbinder: University of Lübeck, Germany S. Köhne: University of Lübeck, Germany M. Mascini: GGZ Noord-Holland-Noord, the Netherlands M. Meewisse: Abate, Centre of Expertise in Anxiety and Trauma, the Netherlands S. Menninga: PsyQ Beverwijk, Parnassia Groep, the Netherlands N. Morina: University of Münster, Germany S.A. Rameckers: University of Amsterdam, the Netherlands K. Thomaes: Sinai Center, the Psychotrauma Expertise Center of Arkin and Amsterdam UMC, location VUmc, the Netherlands C.J. Walton: Centre for Psychotherapy, Hunter New England Mental Health Service, Australia I.G. Wigard: PsyQ Amsterdam, Parnassia Groep, the Netherlands A. Arntz: University of Amsterdam, the Netherlands
Name and contact information for the trial sponsor	Investigator initiated clinical trial; A. Arntz (Principal Investigator), A.R.Arntz@uva.nl; Department of Clinical Psychology, University of Amsterdam (P.O. Box 15933, 1001 NK Amsterdam, the Netherlands).
Role of sponsor	This study is an investigator initiated study. Therefore, the funding body had no role in the design of the study, the collection, analysis and interpretation of the data, and in writing the manuscript.

## Background

Posttraumatic stress disorder (PTSD) can develop after exposure to a traumatic event. PTSD is a chronic and severe mental disorder characterized by four main clusters of symptoms, including re-experiencing the traumatic event, avoidance of trauma-related stimuli, negative feelings or thoughts, and increased arousal [1]. Based on meta-analytic reviews and practice guidelines, trauma-focused cognitive behavior therapy (TF-CBT) and eye movement desensitization and reprocessing (EMDR) are recommended as first-line treatments for PTSD [2, 3]. However, the efficacy of traditional (stand-alone) PTSD treatments for patients with childhood trauma-related PTSD (Ch-PTSD) is more uncertain [4–7]. Individuals with Ch-PTSD are characterized by more complex symptoms, such as interpersonal difficulties, emotional regulation problems, impaired self-concept, and memory and attention difficulties [5, 7–9]. A meta-analysis on psychological treatments for Ch-PTSD found evidence that patients with Ch-PTSD can be treated effectively with trauma-focused treatments [7]. However, research into the effectiveness of treatments for patients with Ch-PTSD is limited as existing studies on the effectiveness of PTSD treatments mainly focus on survivors of adult-onset trauma, resulting in an underrepresentation of survivors of childhood-onset trauma [7, 10].

A recent randomized clinical trial (RCT) (IREM) found promising results for two treatments for Ch-PTSD, namely imagery rescripting (ImRs) and EMDR [11]. ImRs involves the patient recalling the start of the traumatic experience and, subsequently, imagining an intervention that changes the course of events to a more positive outcome aimed at satisfying the needs of the patient [12, 13]. In EMDR, the therapist instructs the patient to focus on a traumatic memory, composed of images, emotions, cognitions, and physical sensations, while the therapist concurrently provides a form of active distraction (e.g., following the back and forth movements of the finger of the therapist) [14]. There are indications that both treatments are effective treatments for patients with PTSD related to childhood trauma. In a meta-analysis on treatments for Ch-PTSD, three RCTs evaluating the effectiveness of EMDR were included [7]. It was found that EMDR was effective in treating PTSD as a consequence of childhood trauma, yielding moderate to high effect sizes. Although ImRs has been less studied to date [15], a recent RCT into the effectiveness of ImRs as a stand-alone treatment for Ch-PTSD concluded that ImRs was effective and highly acceptable [16]. Moreover, in the IREM trial evaluating the comparative effectiveness of EMDR and ImRs, large treatment effects were found for both treatments between baseline and one-year follow-up (i.e., *g* = 2.24 for ImRs and *g* = 1.86 for EMDR on the Clinician Administered PTSD Scale for DSM-5) and treatment dropout was limited to 7.7%. These results were favorable compared to the results found in a meta-analysis on treatments for Ch-PTSD [7]. This meta-analysis found an average effect size for pre-treatment to long-term follow-up (≥ 6 months) of *g* = 1.56 and an average dropout of 22.3%. In discussing the positive outcomes of the treatments in the IREM trial, the authors proposed that the low dropout rate and large symptom reduction might be related to the twice-weekly session frequency of each treatment condition. In contrast, in routine mental health care, trauma-focused treatments are commonly delivered once a week [17]. More insight into the impact of session frequency on treatment outcome is therefore needed.

There are indications that session frequency might be related to the effectiveness of trauma-focused treatments for PTSD. Gutner et al. [17] have examined the association between session frequency and treatment outcome of cognitive processing therapy (CPT) and prolonged exposure (PE) among patients with PTSD. They concluded that a higher session frequency was related to better treatment outcome. In addition, other studies have examined the impact of massed treatments (i.e., daily treatment) compared to weekly treatments for PTSD [18–21]. Results suggest that a more intensive format is similarly effective [18–20] or even more effective [21] compared to treatment once a week. Moreover, the massed treatment leads to a faster reduction in PTSD symptoms. The findings that a higher session frequency is related to better outcomes are consistent with findings based on a meta-analysis on treatments for depression [22] and naturalistic studies across different diagnostic groups (e.g., depression, anxiety disorders, personality disorders) [23, 24]. Furthermore, a RCT among depressive patients demonstrated that delivering treatment twice a week is more effective than delivering treatment only once a week (difference in effect size *d* = 0.55 [25]). However, research into the (causal) effect of session frequency on treatment outcome in PTSD patients is scarce, and there is limited understanding of the mechanisms involved.

Several mechanisms underlying the presumed positive impact of a higher session frequency on treatment outcome in PTSD patients have been hypothesized, including the role of memory and learning, therapeutic alliance, and reducing avoidance [17, 18, 23, 26, 27]. One possible explanation is related to learning processes. Trauma-focused treatments involve the processing of corrective information, which is then stored in memory [12, 28, 29]. New insights might be strengthened by more frequently providing corrective information [26]. This is congruent with previous studies in the field of neurobiology that have shown that the continued survival of neurons, which is realized by their reactivation within a time window of maximum 5 days, is important for learning [30]. This suggests that delivering treatment sessions twice a week (i.e., less than 5 days in between sessions) is more beneficial compared to sessions once a week (i.e., more than 5 days in between sessions). Moreover, Bruijniks et al. [27] hypothesized that a high session frequency might lead to a better recall of the content of the previous sessions, which increases the efficiency and effectiveness of the treatment. In addition, Ehlers et al. [18, 19] pointed out that patients with (Ch-)PTSD have problems with concentration and memory and an intensive treatment format may help patients and therapists to keep the therapeutic material in mind.

An alternative hypothesis is that more frequent sessions have a positive effect on the therapeutic alliance, which in turn affects treatment outcome [23, 27]. It has been proposed that when the contact with the therapist is more frequent, the relationship between the patient and therapist develops more rapidly [27]. In addition, less frequent sessions may attenuate the stability of the therapeutic alliance, because both therapist and patient may feel less connected with each other and less actively involved in the therapy [23]. Research among PTSD patients has shown that the therapeutic alliance is positively related to treatment outcome (e.g., [31–33]). Moreover, the importance of the therapeutic relationship to facilitate change was underscored by Ch-PTSD patients receiving EMDR or ImRs [34].

Finally, more frequent treatment sessions may help PTSD patients to reduce their avoidance of confronting trauma memories [17]. A more intensive treatment format may reduce the opportunity for anticipatory fear or anxiety and avoidance behavior between sessions, which might, in turn, reduce resistance and increase the motivation of patients [26, 35, 36]. In qualitative interviews with Ch-PTSD patients and therapists about their experiences with EMDR or ImRs, some patients and therapists reported that the frequent sessions (i.e., twice-weekly sessions) indeed helped to prevent avoidance behavior and to maintain motivation [34, 37]. In addition, a brief treatment format may help with making the treatment more acceptable to patients since they only need to tolerate distress related to the trauma processing for a short period of time [18, 26].

### Current study

In conclusion, initial evidence suggests that session frequency has an impact on the effectiveness of trauma-focused treatments for PTSD. However, there is a paucity of randomized trials comparing the effects of twice-weekly versus once-weekly treatment sessions, and no trials have examined this issue in Ch-PTSD. This article describes the study design of IREM-Freq, a follow-up study of the IREM study, which compared the effectiveness of twice-weekly EMDR and ImRs for Ch-PTSD [38].

#### Primary objective

The primary objective of the current study is to compare treatment outcomes of twice-weekly versus once-weekly sessions of EMDR and ImRs for adult patients with PTSD related to childhood trauma. We will, therefore, perform an international, multicenter RCT conducted in a routine clinical setting. We hypothesize that both treatments will be more effective when delivered twice a week compared to once a week.

#### Secondary objectives

The IREM-Freq study has several secondary objectives. These include investigating (1) the comparative effectiveness of EMDR and ImRs, (2) whether treatment type (i.e., EMDR versus ImRs) moderates the effect of session frequency, (3) the hypothesized change mechanisms underlying the effects of session frequency on treatment outcome (i.e., memory and learning, therapeutic alliance, motivation, and reluctance and avoidance), (4) the (differential) change mechanisms of EMDR and ImRs, (5) potential predictors of treatment outcome and PTSD severity, (6) the role of emotions, cognitions, and memory in the change in PTSD symptoms, (7) the impact of trauma-focused treatments on learned helplessness, (8) the optimization of treatment selection through identifying patient characteristics that predict treatment response across treatment types (EMDR and ImRs) and treatment frequencies (twice-weekly and once-weekly sessions), (9) the perspectives of patients and therapists on the treatments, (10) the network structure of PTSD symptoms, and (11) sudden treatment gains.

## Methods/design

### Design

**Fig. 1:**
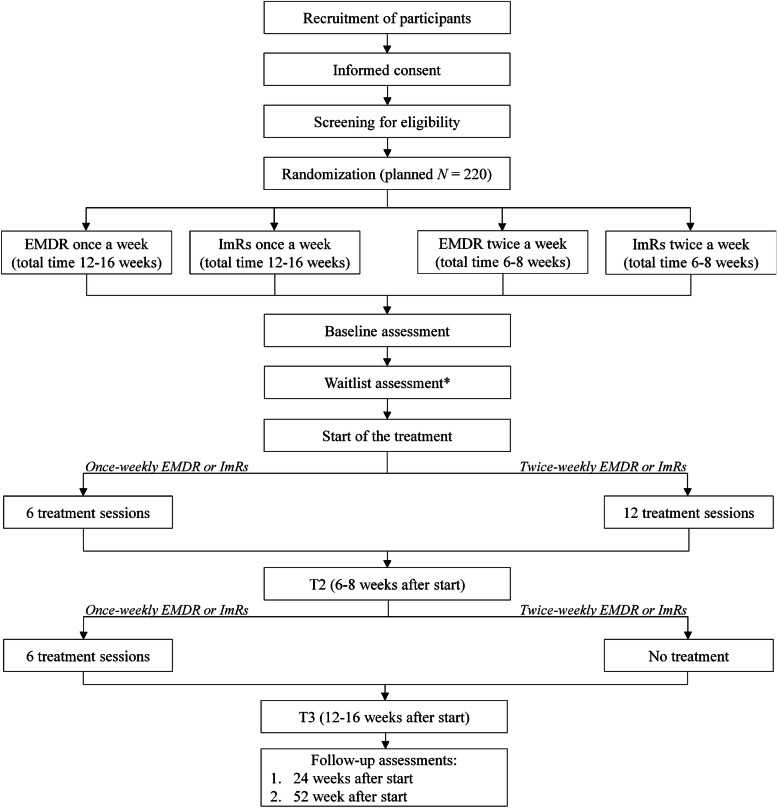
Flow chart of the study design. EMDR = eye movement desensitization and reprocessing; ImRs = imagery rescripting. *A waitlist assessment is included for patients with a waitlist period of more than five weeks before the start of treatment

The study is an international, multicenter superiority RCT with a two by two factorial design, with an equal allocation ratio. The first factor is treatment frequency (twice a week versus once a week), and the second factor is the treatment type (EMDR versus ImRs). The study is registered at the Netherlands Trial Register, part of the Dutch Cochrane Center, with registration number NL6965, and complies with the World Health Organization Trial Registration Data Set. Modifications to the protocol will require a formal amendment to the protocol. The amendments will be examined by the ethics committees of each country and, if approved, included in the trial registration. This trial adheres to the SPIRIT guidelines and methodology [39], see Additional file 1: Appendix A for the SPIRIT checklist. For an overview of the study design, including the recruitment, randomization, assessments, and treatments, see Fig. 1.

### Setting

The study will be carried out in routine clinical settings in eleven mental healthcare centers in the Netherlands (Abate, Enkhuizen; GGZ Noord-Holland-Noord, Heerhugowaard; GGZ Oost Brabant, Helmond; PsyQ, Amsterdam and Beverwijk; and Sinai Centrum, Amstelveen), Germany (University of Lübeck, Lübeck; LMU Munich, Munich; and University of Münster, Münster), and Australia (Centre for Psychotherapy - Hunter New England Mental Health Service, Newcastle and the Sexual Assault Resource Centre, Perth).

### Eligibility criteria

Patients are eligible if they (1) are between 18 and 70 years old; (2) have a primary diagnosis of PTSD as assessed by the Structural Clinical Interview for DSM-5-Clinician Version or Research Version (SCID-5-CV/RV); (3) have experienced the worst traumatic event (defined according to the DSM-5) before the age of 16 and agree that this trauma will be the focus of treatment; (4) have been experiencing PTSD symptoms for longer than 3 months; and (5) have an adequate proficiency in the language of the center or an adequate proficiency in the English language in case the center has English-speaking research assistants and therapists. Patients will be excluded if they (1) have experienced a trauma less than 6 months ago; (2) meet criteria for a current substance use disorder with a moderate or severe severity level (i.e., more than four symptoms), diagnosed with the SCID-5-CV/RV (after 6 weeks of abstinence, participation is possible); (3) fulfill the criteria of a psychotic disorder, diagnosed with the SCID-5-CV/RV; (4) have been diagnosed with the SCID-5-CV/RV with bipolar I disorder; (5) have acute suicide risk; (6) have an IQ below 80; (7) experience serious neurological problems (e.g., dementia); (8) are scheduled to start another form of PTSD treatment; (9) have received PTSD focused treatment within the past 3 months; (10) use benzodiazepine (after 3 weeks of abstinence, participation is possible); or (11) are not able to attend the treatment sessions of both randomization possibilities (i.e., 12 sessions of 90 min within 8 weeks and 12 sessions of 90 min within 16 weeks). In addition, patients will not start treatment unless their medication use has been stable for at least 3 weeks. Finally, patients are not allowed to start any form of psychological treatment or medication between the screening period and 24 weeks after the start of the study treatment (non-PTSD focused supportive treatment may be continued during the screening and waitlist period).

### Sample size

We aim to include 220 participants with about an equal number of participants (*n* = ± 55) in each of the four conditions (i.e., EMDR once a week, EMDR twice a week, ImRs once a week, and ImRs twice a week). We expect a dropout percentage of 10%, which is a conservative estimate given the 7.7% dropout found in the IREM study, the predecessor of the current study [11]. Each site intends to recruit at least 20 patients.

A sample size of *N* = 200 is sufficient to have 94% power to detect a medium effect size (i.e., Cohen’s *d* = .50) between frequency groups (i.e., twice-weekly versus once-weekly sessions of EMDR and ImRs) at post-test or follow-up, assuming equal sample sizes and a two-tailed significance level of *p* < .05, and based on a *t*-test. In addition, the study has 80% power to detect a group difference with an effect size of Cohen’s *d* = .40. Moreover, in case of a significant treatment by frequency interaction, with *N* = 200 power is 80% to detect an effect size of *d* = 0.57 for a comparison of frequency effects within a treatment at a two-tailed significance level of .05 (*t*-test at post-test or follow-up, assuming equal sample sizes of 50 per frequency).

### Procedure: recruitment, informed consent, assessments, blinding, and participant timeline

Patients are recruited from the participating mental healthcare centers. Patients with a (suspected) primary diagnosis of PTSD due to childhood trauma are invited to participate in the screening process to assess for eligibility to participate in the study based on the inclusion and exclusion criteria. The screening process will start after patients have received verbal and written information about the study and signed an informed consent form (see Additional file 1: Appendix B). First, to assess for syndrome and personality disorders, the SCIDs are administered by trained clinicians or research assistants. Second, an interview is conducted to assess the patient’s motivation, availability, and treatment interfering factors. Furthermore, trauma history is assessed using the Life Events Checklist for DSM-5 (LEC-5), including additional items to screen for emotional abuse, emotional neglect, and physical neglect. The LEC-5 is administered once during the screening process to identify the nature and extent of traumatic events. In addition, the worst traumatic event that occurred before the age of 16 and complies with the definition of a traumatizing event according to the DSM-5 (i.e., index trauma) is identified. Finally, the worst traumatic memory of the index trauma and a presently held negative self-referencing belief (i.e., encapsulated belief) are determined. The index trauma, worst memory, and encapsulated belief are used to determine the focus of other measures (i.e., PTSD Checklist for DSM-5 (PCL-5) and the imagery interview).

After the screening assessments are completed, a final check of the inclusion and exclusion criteria is performed by the junior investigator and the patient is randomized by an independent staff member of the sponsor. After randomization, the site investigator of the center is informed of the patient’s allocated treatment condition and searches for a suitable therapist, taking into account the availability of the patient and the preference of the patient for a male or female therapist. At the same time, the baseline assessment (T1) is administered by a research assistant blinded to the patient’s allocated condition. If the time between the start of the treatment and the baseline assessment is more than 5 weeks, an additional assessment (i.e., waitlist assessment) is conducted before the patient starts with the treatment. After the baseline assessment (and, if applicable, the waitlist assessment) is completed and a therapist has been found, the patient starts the treatment. The session frequency of the treatment (i.e., twice a week or once a week) will be revealed to the patient with the invitation for the first session, but the treatment type (i.e., EMDR or ImRs) is not communicated to the patient until the first treatment session.

After treatment has started, patients are re-assessed at four different time points over the course of 1 year: (1) T2: 6 to 8 weeks after the start of the treatment (after six sessions in the once-weekly frequency condition, or after 12 sessions in the twice-weekly frequency condition), (2) T3: 12 to 16 weeks after the start of the treatment (after 12 sessions in the once-weekly frequency condition, or 14 weeks after the start of the treatment in the twice-weekly frequency condition), (3) follow-up 1: 24 weeks after the start of the treatment, and (4) follow-up 2: 52 weeks after the start of the treatment. Patients who discontinue their treatment or deviate from the protocol will be encouraged to continue the assessments. The assessments, including a combination of interviews and computer-based self-report questionnaires, are conducted by a trained research assistant who is blind to the patient’s treatment condition and audio-recorded. Unblinding of research assistants is not permissible. Due to the nature of the interventions, blinding of patients and therapists is not possible. In addition, starting with session 2, the patient and therapist fill out questionnaires before the start of each session to steer the treatment (patient: PCL-5) and to assess potential mediators (patient and therapist: mediator items). Before the fourth and eighth session, the patient and therapist complete an additional questionnaire assessing their memory of the previous session. The questionnaires are completed independently in separate rooms. The study does not involve the collection of biological specimens. An overview of the assessments is presented in Table 1, excluding the candidate predictors of (differential) treatment response that are assessed only once at baseline.

**Table 1:**
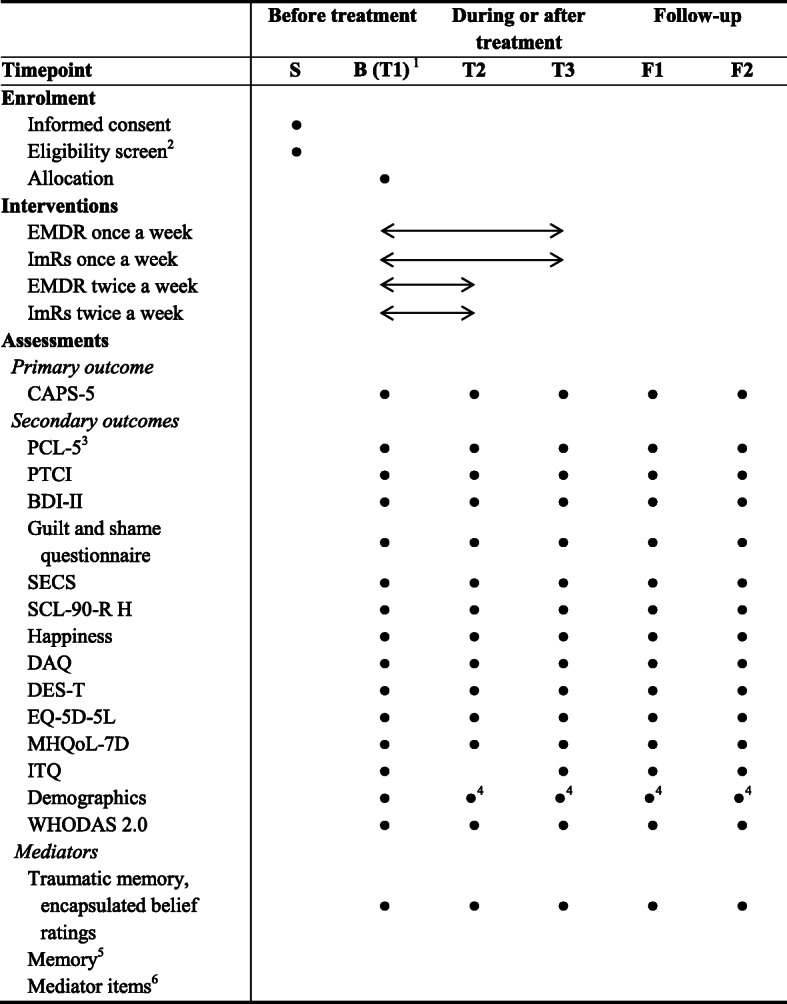
Overview of the assessments

Note. S = screening; B (T1) = baseline assessment; T2 = 6–8 weeks after start of the treatment; T3 = 12–16 weeks after start of the treatment; F1 = 24 weeks after start of the treatment; F2 = 52 weeks after start of the treatment

*BDI-II* Beck Depression Inventory II; *CAPS-5* Clinician Administered PTSD Scale for DSM-5; *DAQ* Depressive Attributions Questionnaire; *DES-T* Dissociative Experiences Scale Taxon; *EMDR* eye movement desensitization and reprocessing; *EQ-5D-5L* 5-level EuroQol 5D version; *ImRs* imagery rescripting; *ITQ* International Trauma Questionnaire; *LEC-5* Life Events Checklist for DSM-5; *MHQoL-7D* Mental Health Quality of Life seven-dimensional Questionnaire; *PCL-5* PTSD Checklist for DSM-5; *PTCI* Posttraumatic Cognitions Inventory; *SCL-90-R H* Symptom Checklist-90-Revised Hostility subscale; *SCID-5-CV* Structured Clinical Interview for the DSM-5 Disorders-Clinician Version; *SCID-5-PD* Structured Clinical Interview for the DSM-5 Personality Disorders; *SECS* Self-Expression and Control Scale; *WHODAS 2.0* WHO Disability Assessment Schedule 2.0

^1^An additional assessment (waitlist assessment) is conducted if patients have a waitlist period of more than 5 weeks before the start of treatment

^2^Consisting of an assessment of the DSM-5 disorders, lifetime exposure to traumatic events, index trauma, traumatic memory, and encapsulated belief

^3^Extended with seven additional items. In addition to the standard assessments, the PCL-5 is also completed before every treatment session, starting with session 2

^4^A brief version of the semi-structured demographic interview including questions about the employment situation, medication use, substance use, and use of psychological treatments other than the study treatment

^5^This test for patients and therapists is assessed before the fourth and eighth treatment session

^6^These items are completed before every treatment session, starting with session 2, by the patient and therapist

Finally, the treatment sessions are videotaped. These recordings are used for supervision and adherence ratings. Within each country, a random sample of treatment sessions will be selected and rated for adherence by trained raters who are blind to condition. A subsample will be rated twice to assess the intraclass correlation coefficient (ICC). The recordings may also be used for secondary investigations (e.g., therapeutic alliance, exploration of effects of micro-techniques).

### Allocation

An independent staff member of the sponsor, not involved in the study, randomizes the patients per center to one of the four conditions (i.e., EMDR once a week, EMDR twice a week, ImRs once a week, or ImRs twice a week) using a computer-generated list. This list was created before the first patient was randomized based on block randomization (*N* = four or eight, with block size randomized) per center, and stratified by gender, to guarantee the balance between conditions per site and over time, and to control for the gender distribution. By randomly varying the block size, the unpredictability of the allocation sequence is enhanced. Only the independent staff member has access to the random list and will keep the list until the end of the research to ensure allocation concealment. The condition will be revealed to the patient with the invitation for the first treatment session (session frequency) and during the first treatment session (treatment type).

### Interventions

#### Format

Treatment consists of a maximum of 12 face-to-face sessions of 90 min. Based on the frequency condition the patient is allocated to, the treatment has a maximum duration of 8 weeks (twice-weekly frequency condition) or 16 weeks (once-weekly frequency condition). In the twice-weekly frequency condition, treatment sessions are offered twice a week with at least 1 day between the sessions. The time between sessions in the once-weekly frequency condition needs to be at least 6 days. Patients that have completed treatment before they reach the maximum of 12 sessions are allowed to complete treatment earlier, but the assessments are conducted at the originally planned assessment times. Early termination of treatment requires approval from the site investigators.

For both treatments, the first session involves scheduling the subsequent sessions (if not already done), introducing the treatment model, and constructing a list of trauma memories to be targeted during treatment. Patients in the ImRs condition receive a pilot rescripting of a minor, non-traumatic experience, to become familiar with the technique. Due to time constraints, there will be no pilot in the EMDR condition. In this condition, patients are prepared for trauma processing in the second session by doing the EMDR procedure preparation. In the subsequent sessions of both EMDR and ImRs, trauma processing of at least one traumatic memory is required in each session. The PCL-5, which will be completed by the patient before each session, can inform the patient and therapist about specific issues that should be addressed during the session (e.g., increase in guilt feelings). After each treatment session, therapists record the duration of the session and the number of traumas that were addressed during the session. Both treatments have been operationalized in treatment manuals.

#### EMDR

EMDR sessions are based on the eight phases of the EMDR standard protocol [14, 40]. The first session, as outlined above, incorporates Phases 1–3, namely history, preparation, and assessment. In addition to developing a list of traumatic experiences, the theory behind EMDR is explained, including describing the adaptive information processing model. A “safe place” is established, and the eye-tracking task is introduced. Based on a traumatic memory taken from the trauma list, the participants identify an image that represents the worst part of the memory. They are then supported to elicit a self-referential negative cognition associated with that image (for example, “I am a failure”). Following, the participant identifies a positive cognition to express what they would want to believe about him or herself when thinking of the image (for example, “I am worthwhile”).

Sessions 2–11 continue to follow Phases 4–8 of Shapiro’s standard protocol (desensitization, installation, body scan, closure, and reevaluation). At the beginning of each session, the participants are asked to comment on any intrusions experienced since the last session. They are then asked to return their focus to the memory that was discussed in the previous session. The participant is asked how the memory is recalled, including associated emotions, body sensations, and the subjective level of distress. Subjective distress is measured on a scale from 0 to 10; 10 being the highest level of disturbance and 0 the minimum. The participant also rates the validity of their positive belief when thinking about the traumatic memory. If the distress is two or less or if the same memory has been processed for two sessions, the therapist then chooses another memory from the trauma list. If not, the therapist continues with the standard EMDR protocol Phases 4–7 (i.e., desensitization, installation, body scan, and closure).

In Session 12 (or earlier if all disturbing memories are processed prior to this), the therapist is instructed to administer the future template. Accordingly, instead of focusing on a past memory, the participant selects a potential future situation that evokes distress, and this is then targeted to overcome any anticipatory anxiety or avoidant behavior.

#### ImRs

ImRs is delivered based on a modified version of the protocol developed by Arntz and Weertman [13]. In short, the therapist helps the patient to imagine a traumatic event and then a helping figure is introduced and changes the script to a better outcome where the needs of the traumatized person are met. Patients are instructed to describe the trauma in the first person, from the point of view of the child, in the present tense. They are guided to identify all sensory information (i.e., what they see, hear, feel, and smell), as well as their thoughts, feelings, and needs. When the traumatic memory and the associated emotions are sufficiently activated, the therapist will move to the rescripting part and introduce the helping figure. Thus, patients do not have to experience the whole trauma with its “hot spot”. In the first six sessions, the therapist is introduced as a helping figure, accompanies the child in the image and intervenes to stop the threat. The therapist turns to the child to ensure that the child’s needs are met (e.g., by comforting, reassurance, or reattribution). In Sessions 7–12, the patient’s adult self enters the image and then rescripts the scenario reporting it from the adult point of view. This is then repeated from the child’s point of view, allowing the child to experience to be protected and cared for by the adult self as well as to ask for any additional support required to ensure its needs are met. Trauma memories are “rescripted” until the patient is satisfied.

#### Therapists, training, and supervision

All therapists in this study are psychologists, psychotherapists, or psychiatrists trained in ImRs, EMDR, or both treatment types. In order to participate, EMDR therapists must have completed a level 1 basic training in EMDR and ImRs therapists were required to complete a basic training course in cognitive behavioral therapy (CBT). In addition, all therapists must have completed an additional 2-day training in EMDR or ImRs for PTSD related to childhood trauma focusing on the treatment protocols as investigated in the study.

Before therapists can start with the treatment, they are required to demonstrate their competence to deliver the treatment with pilot patients. These pilot treatments are evaluated during peer supervision and by the site investigator of the center. During the study, therapists are obliged to attend weekly peer supervision or supervision by an EMDR or ImRs supervisor at their site. Moreover, therapists and site supervisors can consult an expert for questions regarding EMDR (Christopher Lee) and ImRs (Arnoud Arntz). Treatment recordings can be used for supervision. In addition, treatment recordings are also assessed for treatment integrity using adherence scales (EMDR: modified EMDR Therapy Fidelity Rating Scale [41], and ImRs: ImRs Therapist Adherence and Competence Scale [42]).

#### Ancillary and post-trial care

In case of acute crisis during the study, patients may alter their medication or engage in another form of psychological treatment in addition to the study treatment. This will not lead to exclusion from the study. Details of medication use and other psychological treatments next to the study treatment will be recorded.

Following the assessment at 24 weeks after start of the treatment, the therapist will see the patient for an evaluation to determine if more treatment is needed. The type, intensity, and frequency of a potential further treatment will be determined based on the needs of the patient and the possibilities of the center. Details of any further treatment will be recorded. Note that further treatment might focus on other problems than PTSD.

### Coronavirus disease (COVID-19) pandemic

This study is being conducted during the coronavirus disease pandemic. As a consequence of this pandemic, face-to-face treatments can be restricted in mental healthcare centers depending on government and healthcare center policy. Patients who started treatment before the restrictions were executed will receive the remaining treatment sessions via videoconferencing, have a treatment pause, or discontinue with treatment because of no possibilities for videoconferencing. These patients will be excluded from the study as we will only include patients who received treatment face-to-face, so as not to introduce a confounding variable. The treatment will be postponed for patients for whom the treatment had not started or who had received only one treatment session. Treatment will be resumed when face-to-face treatment is permitted again, possibly with the use of a plexiglass barrier between the patient and therapist. These patients will be included in the study. Assessments will be conducted via phone or videoconferencing, and questionnaires will be completed by participants at home if face-to-face assessments are not allowed.

### Measures

The instruments include screening, primary outcome, and secondary outcome measures. In addition, potential mediators and predictors of treatment outcome are also assessed. All instruments that had not been available in the languages of the participating sites were translated. The translation versions were checked for consistency with the original version.

#### Screening

##### Mental disorders

Syndrome disorders, according to the DSM-5 criteria, are assessed using the SCID-5-CV [43] or SCID-5-RV [44]. Personality disorders are assessed with the SCID for DSM-5 personality disorders (SCID-5-PD [45]) or SCID for Axis II disorders (SCID-II [46]). The SCID-5-CV is extended with additional disorders and modules from the SCID-5-RV, including gambling disorder, specific phobia, body dysmorphic disorder, intermittent explosive disorder, feeding and eating disorders, somatic symptom and related disorders, and sleep disorders. Additional file 1: Appendix C offers an overview of all syndrome disorders that are assessed with the extended SCID-5-CV. Since the German SCID-5-CV/RV was not yet available during the start of the study, the SCID for DSM-IV Axis I disorders (SCID-I [47]) was assessed temporarily in the German centers. In order to converge to the DSM-5 criteria, missing disorders (e.g., gambling disorder, insomnia disorder) were added to the SCID-I, time periods were changed if needed, and additional items were added to the PTSD module. The SCID-I and SCID-II have demonstrated satisfactory psychometric properties (e.g., [48–50]). Based on an initial psychometric evaluation, the SCID-5-CV and SCID-5-PD have shown adequate psychometric properties [51, 52].

##### Lifetime trauma exposure, index trauma, traumatic memory, and encapsulated belief

The extended self-report version of the Life Events Checklist for DSM-5 (LEC-5 [53]) is used to screen for lifetime exposure to traumatic events. For the purposes of the study, we have added traumas, including exposure to emotional abuse, emotional neglect, and physical neglect (see Additional file 1: Appendix D). Traumatic events that have been endorsed by the participant will be evaluated by the interviewer to ascertain the number of times an event occurred and the age of the participant. In addition to the screening of traumatic events, the index trauma, worst traumatic memory of the index trauma, and encapsulated belief are identified in direct discussion with the participant.

#### Primary outcome

##### PTSD severity

The primary outcome is the change in the severity of the DSM-5 PTSD symptoms between the baseline and follow-up 1 assessments, assessed with the Clinician Administered PTSD Scale for DSM-5 (CAPS-5 [54]). The CAPS-5 is a structured interview consisting of 30 5-point Likert scale items ranging from 0 (absent) to 4 (extreme/incapacitating) rating the 20 DSM-5 PTSD symptoms in the past month. In the current study, the CAPS-5 assesses responses to all traumatic events that have been identified with the LEC-5. The CAPS-5 has demonstrated good reliability and validity [55–57].

The assessment period of the CAPS-5 (i.e., past month) at the T3 assessment overlaps with the treatment period in the once-weekly frequency condition. Therefore, we chose to focus on change between baseline and follow-up 1 instead of baseline and T3 since the follow-up 1 assessment takes place several weeks after treatment has finished and does not overlap with the treatment period (i.e., administered 8–12 weeks after treatment has finished in the once-weekly frequency condition and 16–18 weeks after treatment has finished in the twice-weekly treatment condition).

#### Secondary outcomes

##### Self-reported PTSD symptoms and trauma-related feelings

Self-reported PTSD symptoms are measured using the PTSD Checklist for DSM-5 (PCL-5 [58]). The PCL-5 consists of 20 5-point Likert scale items ranging from 0 (not at all) to 4 (extremely). We created two versions of the PCL-5; one version assessing self-reported PTSD symptoms with respect to the index trauma and the other one for all traumatic experiences except of the index trauma. The time frame was changed from the past month to the past week. Psychometric properties of the PCL-5 were found to be good [59–61].

Seven single-item scales were added to the PCL-5 [38, 62], see Additional file 1: Appendix D. Six items were included to assess trauma-related feelings (i.e., feelings of shame, anger, guilt, disgust, sadness, and anxiety) with respect to the index trauma and for all traumatic experiences except of the index trauma. An additional item was included to assess feelings of happiness in the past 7 days, for secondary analysis purposes. The additional items will not be used for computation of the total PCL-5 score.

##### PTSD-related cognitions

The Posttraumatic Cognitions Inventory (PTCI [63]) is a 33-item questionnaire measuring trauma-related cognitions across three domains (i.e., negative cognitions about self, negative cognitions about the world, and self-blame). The items are rated on a 7-point Likert scale ranging from 1 (totally disagree) to 7 (totally agree). The PTCI has demonstrated satisfactory psychometric properties [63].

##### Depressive symptoms

Depressive symptoms during the last 2 weeks are measured by the Beck Depression Inventory II (BDI-II [64]). The BDI-II consists of 21 items containing a list of four statements with regard to a particular symptom of depression. The BDI-II has shown good psychometric properties [65].

##### Guilt and shame

An eight-item questionnaire was developed based on the Personal Feelings Questionnaire 2 (PFQ2 [66]) and the Adapted Shame and Guilt scale (ASGS [67]) to assess feelings of guilt and shame in the past 4 weeks (see Additional file 1: Appendix E [68]). The items are rated on a 5-point Likert scale ranging from 0 (never) to 4 (daily). A validation study is currently running.

##### Anger

Anger is measured using the Self-Expression and Control Scale (SECS [69]) and the Hostility subscale of the Symptom Checklist-90-Revised (SCL-90-R [70, 71]). The SECS measures anger expression and anger control and consists of 40 items scored on a 4-point Likert scale ranging from 1 (almost never) to 4 (almost always). Previous research has demonstrated good psychometric properties [69, 72]. The Hostility subscale of the SCL-90-R contains six 5-point Likert scale items ranging from 0 (not at all) to 4 (extremely) and assesses anger related thoughts, feelings, and actions. The subscale demonstrated satisfactory validity and reliability [73, 74].

##### Happiness

Happiness is assessed by a single 7-point Likert scale item ranging from 1 (completely unhappy) to 7 (completely happy) which measures general happiness in the weeks prior to the assessment [75]. Reliability and validity ranged from good to excellent [75].

##### Depressive attributions

The Depressive Attributions Questionnaire (DAQ [76]) measures depressogenic attributions. The DAQ consists of 16 items measuring helplessness and negative internal, stable, and global attributions. The items can be rated on a 5-point Likert scale ranging from 0 (not at all) to 4 (very strongly). Earlier research has demonstrated good psychometric properties [76, 77].

##### Dissociative experiences

Dissociative experiences are assessed with the Dissociative Experiences Scale Taxon (DES-T [78]), a brief version of the DES [79]. The DES-T contains eight items rated on an 11-point Likert scale ranging from 0% (never) to 100% (always) at 10% intervals. Psychometric properties of the DES-T are satisfactory [80].

##### Quality of life

Quality of life is measured using the 5-level EuroQol 5D version (EQ-5D-5L [81]) and the Mental Health Quality of Life seven-dimensional Questionnaire (MHQoL-7D [82]). The EQ-5D-5L assesses five health state dimensions (mobility, self-care, usual activities, pain/discomfort, and anxiety/depression), whereby each dimension is divided into five severity levels ranging from no problem to extreme problems. The EQ-5D-5L has shown to be a valid and reliable measure [83].

The MHQoL-7D was recently developed and assesses quality of life in people with mental health problems. The MHQoL-7D contains seven quality of life domains (self-image, independence, mood, relationships, daily activities, physical health, and hope) divided into four severity levels and a visual analog scale (VAS) assessing patient’s psychological well-being. Currently, a study into the psychometric properties of the MHQoL-7D among PTSD patients is running. The MHQoL-7D will only be included in the analysis if it is demonstrated to be a psychometrically sound instrument. As the recruitment of patients started before the MHQoL-7D was available, the quality of life instruments are assessed in a subset of the patients.

##### Complex PTSD symptoms

The 18-item International Trauma Questionnaire (ITQ [84]) measures complex PTSD (CPTSD) symptoms according to the diagnostic formulation published in the 11th revision of the International Classification of Diseases (ICD-11 [85]). To minimize participant burden, only nine items measuring the three Disturbances in Self-Organization (DSO) clusters (i.e., affective dysregulation, negative self-concept, and interpersonal problems) and functional impairments related to DSO symptoms are administered. The combined data of PCL-5 scores and DSO scores will provide dimensional scoring, as well as diagnostic information (i.e., diagnosis of PTSD or CPTSD based on the ICD-11 diagnostic rules). The ITQ has demonstrated adequate internal reliability and validity [84, 86]. Recruitment of patients had commenced before the ITQ was included and, therefore, the ITQ is assessed in a subset of the patients.

##### General, social, and societal functioning

General, social, and societal functioning is assessed by the 12-item World Health Organization Disability Assessment Schedule 2.0 (WHODAS 2.0) interview version [87]. The WHODAS 2.0 assesses experienced difficulties in six major life domains (i.e., cognition, mobility, self-care, getting along, life activities, and participation) in the past 30 days on a 5-point Likert scale ranging from 1 (none) to 5 (extreme or cannot do). The WHODAS 2.0 has shown good psychometric properties [87].

##### Demographics

General patient characteristics (e.g., age, ethnicity, marital status, educational level) will be collected using a semi-structured demographic interview. Additional information will be collected with regard to patient's employment situation, medication use, substance use, and use of psychological treatments other than the study treatment (i.e., ImRs or EMDR).

#### Mediators

Potential mechanisms underlying the presumed effect of session frequency on treatment outcome include memory, learning, therapeutic alliance, motivation, reluctance, and avoidance.

##### Memory

Memory of the previous session is assessed by a recently developed memory test (see Additional file 1: Appendix F) in which patients and therapists are asked to think back to what happened in the previous session and to describe as precisely as possible the most important issues of the previous session.

##### Mediator items

The mediator items have been specifically developed for the study (see Additional file 1: Appendix G) and include nine 100 mm VAS items assessing memory and learning (two items: memory of the previous session and time to process), therapeutic alliance (three items: working alliance, connection, and support), motivation (one item), and reluctance and avoidance (three items: reluctance to participate, difficulty of the treatment, and optimism about the final outcome of the treatment). In addition to these nine items, one additional item is filled out by the patient about life events that may interfere with the treatment.

##### Traumatic memory and encapsulated belief ratings

Traumatic memory vividness, distress and valence, and credibility of the encapsulated belief are potential mechanisms of change of EMDR and ImRs [38] and assessed by a semi-structured imagery interview [88–91]. Participants are asked to recall the worst traumatic memory of the index trauma, identified during the screening procedure. After the image of the memory has been elicited, the vividness and controllability of the memory and the degree of distress related to the memory are rated on a 100 mm VAS. In addition, the valence of the traumatic memory (e.g., anger, sadness, shame) is evaluated on eight 5-point Likert scale items ranging from 0 (not at all) to 4 (extremely). Finally, participants rate to what extent they believe their encapsulated belief is true. This procedure has been used in previous research [38].

#### Predictors

Candidate predictors of (differential) treatment response were selected based on the literature, expert consensus, and preliminary analyses of the IREM study [11]. The predictors are assessed during the baseline assessment. Only the measures that are not part of the screening and secondary outcome measures are briefly described below.

##### Childhood maltreatment

The Childhood Trauma Questionnaire-Short Form (CTQ-SF [92]) assesses the severity of five types of childhood maltreatment (i.e., emotional abuse, physical abuse, sexual abuse, emotional neglect, and physical neglect). The CTQ-SF consists of 28 items rated on a 5-point Likert scale ranging from 1 (never true) to 5 (very often true). Several studies have demonstrated satisfactory psychometric properties (e.g., [92–94]).

##### Experiential avoidance

The Brief Experiential Avoidance Questionnaire (BEAQ [95]) assesses experiential avoidance across six different domains (i.e., behavioral avoidance, distress aversion, suppression, procrastination, repression/denial, and distress endurance). The BEAQ consists of 15 6-point Likert scale items ranging from 1 (strongly disagree) to 6 (strongly agree) and has demonstrated satisfactory psychometric properties [95].

##### Readiness to change

Readiness to change is measured by the subscales Contemplation and Action of the 24-item version of the University of Rhode Island Change Assessment (URICA [96–98]). Each subscale consists of six items that are scored on a 5-point Likert scale ranging from 1 (strongly disagree) to 5 (strongly agree). Both subscales have demonstrated satisfactory internal consistency [99].

##### Social problems

Social problems (e.g., housing problems, unemployment, social isolation) are assessed in direct discussion with the participant by using the social problems list, derived from the Improving Access to Psychological Therapies (IAPT) program [100].

##### Openness to experience

The openness to experience subscale of the Big Five Inventory (BFI [101]) is a 10-item scale that measures openness to experience. Each item can be rated on a 5-point Likert scale ranging from 1 (disagree strongly) to 5 (agree strongly). The subscale has demonstrated satisfactory validity and reliability [102].

##### Working memory

An adapted version of the subtask Doing sums of the Groningen Intelligence Test (GIT [103]) is used as a proxy for working memory. This task involves the correct completion of as many adding sums as possible in 1 min. The test-retest reliability of the subtask Doing sums was satisfactory in previous research [104].

##### Willingness to talk about traumatic experiences

Willingness to talk about traumatic experiences is assessed by two 100 mm VAS items specifically developed for the study (see Additional file 1: Appendix H).

##### Schema mode ratings: detached protector and suspicious overcontroller

Two schema modes (i.e., detached protector mode and suspicious overcontroller mode) are measured using two subscales of the Schema Mode Inventory 1 and 2 (SMI [105, 106]). Both subscales consist of nine 6-point Likert scale items ranging from 1 (never or almost never) to 6 (all of the time) and demonstrated acceptable psychometric properties [107].

##### Mental imagery capacity

The Gordon Test of Visual Imagery Control (TVIC [108]) is used to assess mental imagery capacity. This TVIC consists of 12 3-point Likert scale items (yes, no, and unsure) measuring the ability to visualize and manipulate mental images. In addition to the Likert scale, we also measure the time it takes the participant to visualize the scenes. Finally, we have added two 100 mm VAS items measuring how well the participant sees the mental scenes that were described and how difficult it was for the participant to visualize the different scenes (see Additional file 1: Appendix D). The TVIC has shown acceptable to satisfactory validity and internal consistency [109–111].

##### Avoidance of shame and guilt

The extent to which people tend to avoid sharing issues they feel ashamed about and issues they feel guilty about is assessed by two 100 mm VAS items specifically developed for the study (see Additional file 1: Appendix I).

##### Attachment style

Participant’s general attachment style is measured by the Relationship Structures Questionnaire (ECR-RS [112]), a brief version of the Experience in Close Relationships-Revised (ECR-R [113]). The ECR-RS consists of nine 7-point Likert scale items ranging from 1 (strongly disagree) to 7 (strongly agree) and assesses attachment-related avoidance and attachment-related anxiety. The ECR-RS has demonstrated good psychometric properties, comparable to the ECR-R [112].

##### Frustration tolerance

Frustration tolerance is assessed by the Frustration tolerance scale of the Personality Data Form (PDF [114]). This subscale consists of 13 3-point Likert scale items ranging from 1 (often) to 3 (seldom). Shorkey and Sutton-Simon [115] have found initial evidence for the reliability and validity of the PDF.

##### Rumination

Rumination is measured by the Perseverative Thinking Questionnaire (PTQ [116]). The PTQ consists of 15 5-point Likert scale items ranging from 0 (never) to 4 (almost always) and assessing the frequency of dysfunctional, repetitive thinking. The PTQ has demonstrated good psychometric properties [116, 117].

### Statistical analyses

The statistical analyses will be performed according to the intention-to-treat principle. Change in the primary and secondary outcome measures and the relative effectiveness of the two frequency conditions (twice-weekly frequency condition versus once-weekly frequency condition) will be analyzed using mixed regression analyses. By using mixed regression analysis, all available data will be used, and dependencies among observations nested within individuals will be taken into account. Appropriate forms of the mixed regression models will be selected, based on the variable type (e.g., scale, count, dichotomous) and taking into account the distribution of the residuals. To assess the comparative effectiveness of EMDR and ImRs and whether treatment type moderates the effect of session frequency, additional terms will be added to the model. In addition to the primary analyses based on the intention-to-treat principle, a completers analysis focused on the change in the primary outcome measure will be conducted by excluding patients who discontinued treatment or deviated from the protocol (e.g., duration of more than 16 weeks in the once-weekly frequency condition, other psychological treatment next to the study treatment, unstable level of medication during the treatment period). No interim analyses are planned. Finally, to examine underlying change mechanisms of session frequency (i.e., memory and learning, therapeutic alliance, motivation, and reluctance and avoidance), advanced mediation analysis will be performed (e.g., mixed regression for Granger Causality models [118]).

The additional substudies will be analyzed using different statistical methods. To study (differential) mechanisms of change of EMDR and ImRs (i.e., the strength of the encapsulated belief, and the vividness and valence of the traumatic memory of the index trauma), mediation analysis will be conducted using Granger Causality models. In addition, mixed regression analysis will be used to investigate whether comorbid personality disorder psychopathology and trauma characteristics are related to treatment effects. The role of emotions, cognitions, memory, and learned helplessness in altering PTSD symptoms over time will be examined using advanced mediation analyses (e.g., mixed regression for Granger Causality models [118]). Furthermore, a two-step approach will be used to determine the optimal treatment type (EMDR versus ImRs) and frequency (twice-weekly versus once-weekly) for a particular patient. First, we will examine which of the candidate predictors predict what treatment and which frequency will be more beneficial. Different variable selection approaches can be applied, including bootstrapping procedures [119], a Random Forest machine learning approach [120], and the domain approach outlined by Fournier et al. [121]. Second, a prediction model will be built based on the identified predictors to generate individual treatment recommendations geared to predict the primary outcome measure (i.e., PTSD severity). Since statistical methods for the selection of variables and generating individual treatment recommendations are still in development, we will select the optimal methods at the time of the analyses. Finally, to investigate the network structure of (complex) PTSD symptoms at baseline and changes in the network structure of patients as a consequence of treatment, network analysis and longitudinal comparisons between networks will be conducted (e.g., [122, 123]).

### Additional substudies

#### Change mechanisms of EMDR and ImRs

This study aims to test the proposed (differential) mediators of EMDR and ImRs (i.e., valence and vividness of the traumatic memory, and the strength of the encapsulated belief). We hypothesize that (1) the effect of change in the encapsulated belief on PTSD symptom change is stronger for ImRs and (2) the effect of change in memory vividness on PTSD symptom change is stronger for EMDR. As memory valence changes have been observed in both treatments and thus findings are mixed [124–126], we will not make specific predictions for this mediator. In order to test the causality of effects, which is currently unknown, we will also test the reverse relationships. As an exploratory analysis, we might also conduct a model selection procedure, but the main focus lies on testing the predictors in separate models.

#### Predictors of treatment outcome and PTSD severity: personality disorder psychopathology and trauma characteristics

The aim of this study is to examine whether comorbid personality disorder psychopathology and trauma characteristics are related to (differential) treatment outcome. In clinical practice, patients with comorbid personality disorder psychopathology are often assumed to benefit less from treatment focused on their syndrome disorder compared to patients without comorbid personality disorder psychopathology [127, 128]. However, there is mixed evidence regarding the effect of comorbid personality disorder psychopathology on treatment outcome in PTSD patients [129]. In addition, mixed results were also found for the effect of trauma characteristics, including type and severity of childhood trauma [129].

Trauma characteristics may also play a role in the prediction of PTSD symptoms, which in turn might predict differential treatment response. Whilst sexual and physical abuse in childhood can give rise to PTSD [130], several studies have suggested that other types of childhood maltreatment (e.g., emotional abuse or neglect) can also result in PTSD although not officially qualifying as traumatic experiences according to the DSM-5 [131–133]. Therefore, one aim will be to explore the unique relationships between childhood abuse and neglect and PTSD severity. A model fit procedure will also be conducted to test which of these types remain in a final model.

#### Role of emotions

This study will investigate patterns of change over time of emotions (i.e., fear, shame, guilt, sadness, disgust, and anger) and PTSD symptoms between sessions and assessments. Temporal relations between emotions and PTSD symptoms will be examined. More specifically, we will examine (1) whether changes in emotions precede changes in PTSD symptoms, (2) whether changes in PTSD symptoms predict changes in emotions, and (3) whether there is a reciprocal relationship between changes in emotions and PTSD symptoms.

#### Role of cognitions

Changes in trauma-related cognitions have been found to precede subsequent changes in PTSD symptoms during CPT [134]. In this study, we will examine if changes in trauma-related cognitions (measured with the PTCI) will also precede and predict changes in PTSD symptoms in EMDR and ImRs and, therefore, are an important underlying mechanism of change in PTSD treatment in general beyond CPT.

#### Memory of previous session

A possible working mechanism of the effect of session frequency on treatment outcome is the amount of episodic memory details of the previous session. Rich episodic memory details and vivid re-experiencing of these details have been shown to quickly decay within the scope of days [135, 136]. Repeatedly activating a memory conserves its unique properties and long-term maintenance [137]. Therefore, this study aims to investigate if the effect of session frequency on treatment outcome is mediated by the amount of details patients and therapists remembered and retrieved from the previous session.

#### Learned helplessness

Learned helplessness refers to a pattern of thoughts and behavior instigated by repeated or intense, uncontrollable adverse events, that increases the likelihood of psychopathology, especially depression [138]. This study aims to build on earlier analyses investigating the impact of trauma-focused treatments on levels of learned helplessness [139]. Further, the role of learned helplessness as a possible mediator of change, especially for participants who have a comorbid depression diagnosis, will be investigated.

#### Prediction of (differential) treatment response

Patients with PTSD vary substantially in their response to trauma-focused treatments [140]. More insight into factors associated with treatment outcome in PTSD is therefore needed. The aim of this study is to optimize treatment selection by examining patient characteristics that predict (differential) treatment response across treatment types (EMDR and ImRs) and treatment frequencies (twice-weekly and once-weekly).

#### Perspectives of patients and therapists

Qualitative studies will focus on the perspectives of patients and therapists on the treatments. These studies will focus on, among others, the experiences with the frequency of the sessions and the mechanisms of change in ImRs. In-depth interviews will be performed with a subsample of the study population, including patients and therapists.

#### Network models of PTSD symptoms

The network approach is an alternative approach to conceptualize mental disorders [141]. Network analysis will, therefore, be performed to gain more insight into the network structure of (complex) PTSD symptoms. More specifically, this study aims to investigate the cross-sectional structure of (complex) PTSD symptoms (e.g., connectivity, centrality) of patients before the start of the treatment [142], as well as its change during the course of treatment [143].

#### Sudden gains and treatment outcome

A sudden gain is defined as a large improvement in an outcome variable experienced by a patient between two consecutive sessions. Research on sudden gains in treatments for PTSD suggests that they are positively associated with treatment outcome [144]. However, there is a lack of knowledge about what contributes to sudden gains in treatment of PTSD as well as the role that treatment frequency may play. We hypothesize that (1) therapeutic alliance, memory and learning, and reluctance and avoidance predict sudden gains, (2) the occurrence of sudden gains in the twice-weekly sessions is higher than in the once-weekly sessions, and (3) sudden gains predict treatment outcome.

### Oversight and monitoring

#### Data monitoring and management

The study is guided by the study board, which acts as the steering committee. The study board is composed of the principal investigator, co-principal investigator, junior investigators, and site investigators (see Additional file 1: Appendix J for the names, roles, and responsibilities of the study board members). The study board meets every month and reviews the progress of the study. In addition, final decisions on changes to the protocol, publications, and reporting will be made by the study board. Decisions will be made in consensus or, if necessary, by a majority vote of the study board. Moreover, publication agreements are being made in the study board meetings.

The study will not be audited by independent auditors because of limited study resources. However, to preserve the integrity of the trial, trial processes and documents will be reviewed by the study board members. In addition, there is no data monitory committee as the study involves minimal risk. Both interventions are part of regular care and effective and safe for patients with Ch-PTSD [11]. The data will be monitored for consistency and validity (e.g., check for errors, range checks, missing values) by the junior investigator. Moreover, the data and statistical code will be shared with the study board members.

#### Adverse event reporting and harms

Adverse events (AEs) are defined as any undesirable experience occurring to a subject during the study, whether or not considered related to the trial procedure or intervention. A serious adverse event (SAE) is any untoward medical occurrence or effect that results in death, is life threatening, requires (prolonged) hospitalization, and/or results in persistent or significant disability. All (S)AEs reported by the participant or observed by the researchers or therapists will be recorded. Events that meet the criteria of a SAE will be reported to the junior investigator and principal investigator within 24 h after coming to notice of the site investigator. The relatedness of a SAE to the trial procedure or intervention will be determined. Every month, the junior investigator checks with the site investigators if all (S)AEs have been reported. The final trial report will include a description of the SAEs, although no formal hypothesis testing will take place as low rates are expected [11].

#### Data storage and confidentiality

Data collection is supported by a web tool, Lotus, developed by the University of Amsterdam, which helps researchers managing longitudinal research. Data is processed by Lotus via the secure online survey software Qualtrics [145] with a unique identifier for each participant (i.e., pseudonym). The data is stored on a secure server of the University van Amsterdam accessible only to authorized researchers. A list of the pseudonyms and personal information of the participants within a healthcare center is securely stored at the center and only accessible for the site investigator and research assistant of the center.

#### Data dissemination

The results of the study will be disseminated in scientific journals and presentations at scientific conferences, regardless of the direction or magnitude of effects. Clinicians will be informed by presentations, books, and chapters describing the treatments, training, and supervision. Participants will receive the final trial report on request. In addition, participants receive a report with their results on several outcome measures during the evaluation after 24 weeks after start of the treatment. The participant-level dataset will not be publicly available because it could contain information that compromises the anonymity of participants. The data and statistical code supporting the findings of the final trial report will be available on reasonable request and will comply with the EU general data protection regulation.

The study board members will qualify for (co-)authorship of the publication of the final trial report. The study board members and employees of the trial investigators’ institutions can qualify for (co-)authorship of publications on additional investigations, but only if they fulfill the American Psychological Association criteria for authorship. The current agreement on the planned publications is available on request from the corresponding author.

## Discussion

This article described the study protocol of an international, multicenter RCT comparing twice-weekly versus once-weekly sessions of EMDR and ImRs for patients with PTSD related to childhood trauma. The primary aim of the study is to gain insight into potential differences in outcome of trauma-focused treatments when offered twice a week compared to once a week. Moreover, we aim to elucidate *how* session frequency impacts the effectiveness of the treatments by testing a set of hypotheses about a possible frequency effect. In addition, we will examine the comparative effectiveness of EMDR and ImRs and whether treatment type moderates the effect of session frequency. Finally, several additional investigations will be conducted to broaden our understanding of how the treatments work and variables that affect treatment outcome.

This trial provides the opportunity to examine the effectiveness of two promising trauma-focused treatments for Ch-PTSD—EMDR and ImRs—with regard to session frequency and mechanisms of change. More insight into the optimal session frequency could lead to improved treatment outcomes and less dropout, and in turn to a reduction of direct and indirect healthcare costs. The finding that a higher session frequency is superior to a lower session frequency would have important implications for clinical practice. In current clinical practice, it is common that treatment sessions are scheduled once per week or even less frequently [17]. It may not always be feasible for patients and therapists to schedule treatment sessions twice a week because of other commitments. Practical demands such as administrative tasks and too much clinical work may prevent therapists from scheduling more frequent sessions [146]. However, if treatment outcomes are indeed improved by increased session frequency, we would encourage therapists to consider scheduling twice-weekly sessions in order to optimize (cost-)effectiveness of EMDR and ImRs.

In addition, this study will provide insight into the working mechanisms of EMDR and ImRs and the mechanisms underlying the effects of session frequency. Studying mechanisms of change helps to identify core ingredients of interventions, which is important for improving the treatments [147]. Moreover, potential variation in outcomes between Ch-PTSD patients will be investigated. The main findings of this RCT refer to the effects of the treatments on average and do not address the potential heterogeneity in treatment effects. However, individual differences among patients may affect response to treatment type and frequency. For example, it is conceivable that for some patients with specific characteristics treatment once per week is indicated, while for the majority of patients twice a week is a better choice. Investigating the variation in outcomes between Ch-PTSD patients can help patients and therapists to select the optimal treatment, which in turn will lead to improved treatment outcomes [148]. Finally, this study will contribute to the evidence of stand-alone trauma-focused treatments for patients with PTSD related to childhood trauma. If we find that EMDR and ImRs are indeed effective, this study might help reduce the barriers to engage in stand-alone trauma-focused treatments for patients with Ch-PTSD.

The IREM-Freq study has several strengths. First, only a few exclusion criteria are applied in this study. Most trials on Ch-PTSD exclude patients with dissociative symptoms and personality disorders [5, 10, 149]. By evaluating the effectiveness of twice-weekly and once-weekly EMDR and ImRs in a broad and representative group of patients, the generalizability of the findings to routine clinical practice is enhanced. In addition, the multicenter and international design of the trial will also increase generalizability.

Second, the study is conducted by a research group with different areas of allegiances and expertise. This will prevent a potential effect of researcher allegiance on the results [150], which has been described as one of the biggest threats to internal validity [151]. Third, this trial does not only focus on change in PTSD symptoms, but other symptoms common in Ch-PTSD patients (e.g., shame, guilt, anger, dissociation, and functional impairment) are also included. It can, therefore, be examined whether EMDR and ImRs are effective for a wider range of symptoms.

A final strength of this study is the methodological quality. According to Ehring et al. [7], studies on the effectiveness of treatments for Ch-PTSD often lack methodological quality and more rigorous approaches are needed. This trial includes a multi-method assessment approach (i.e., self-report measures and semi-structured interviews), an assessment of treatment integrity, manualized treatments, and a long-term follow-up. Moreover, the semi-structured interviews are administered by independent, trained research assistants to control for interviewer bias. In addition, the outcome and presumed mechanisms of change of session frequency are assessed every treatment session, allowing us to examine concurrent as well as temporal relationships using state-of-the-art statistical analysis methods [152].

There are several limitations to this study that should be considered when evaluating the results. First, the study treatments are not compared to other trauma-focused treatments, including TF-CBT approaches such as imaginal/prolonged exposure (IE/PE) and cognitive (processing) therapy (CT/CPT) for PTSD. In addition, this study does not include a randomized waitlist control group, which can affect internal validity. When patients improve in all treatment conditions and there are no differences between conditions, it cannot be ruled out that non-treatment-specific factors (e.g., attention, time) have caused the improvements. On the other hand, the study does include an additional assessment if there is a waitlist period of at least 5 weeks (i.e., waitlist assessment). If there are enough patients with a waitlist assessment, we will test the effects of active treatment compared to naturalistic waitlist, assuming that the duration of the waitlist period is nonbiased. Moreover, including a control group receiving no treatment above the natural wait at the participating site would increase patients’ resistance to participate in the trial, which would result in selection bias.

Second, the study is not powered to reliably detect (≥ 80% power) small effects of session frequency, or the interaction between frequency and treatment method. Finally, the training requirements for therapists differ between the treatments types and there is no budget available for intensive supervision by experts outside the treatment centers. However, experts in the field of EMDR (Christopher Lee) and ImRs (Arnoud Arntz) are available for consultation. Moreover, these conditions reflect clinical practice and, consequently, enhance generalizability of the findings to “real-world” settings.

In conclusion, this study is the first to compare treatment outcomes of twice-weekly versus once-weekly sessions of EMDR and ImRs for adult patients with PTSD related to childhood trauma. Research into the effect of session frequency on treatment outcome in Ch-PTSD patients is highly needed since there are indications that session frequency is related to the effectiveness of trauma-focused treatments for PTSD. Moreover, by investigating underlying mechanisms and predictors of (differential) treatment outcome, we will gain more insight into for whom a treatment works and how. This study will, therefore, significantly extend our knowledge on trauma-focused treatments for Ch-PTSD and, more specifically, the role of session frequency.

## Trial status

Recruitment has started in June 2018 and is still ongoing. The estimated completion date is April 2022. Protocol version 2.1 is currently active.

## Supplementary Information

**Additional file 1.**

## Data Availability

Not applicable.

## Abbreviations

ASGS: Adapted Shame and Guilt scale
BDI-II: Beck Depression Inventory II
BEAQ: Brief Experiential Avoidance Questionnaire
BFI: Big Five Inventory
CAPS-5: Clinician Administered PTSD Scale for DSM-5
Ch-PTSD: Adults with childhood trauma-related PTSD
CPT: Cognitive processing therapy
CPTSD: Complex PTSD
CT: Cognitive therapy
CTQ-SF: Childhood Trauma Questionnaire-Short Form
DAQ: Depressive Attributions Questionnaire
DES-T: Dissociative Experiences Scale Taxon
DSM-5: Diagnostic and statistical manual of mental disorders, fifth edition
DSO: Disturbances in Self-Organization
ECR-R: Experience in Close Relationships-Revised
ECR-RS: Relationship Structures Questionnaire
EMDR: Eye movement desensitization and reprocessing
EQ-5D-5L: 5-Level EuroQol 5D version
GIT: Groningen Intelligence Test
IAPT: Improving Access to Psychological Therapies
ICD-11: 11th revision of the International Classification of Diseases
IE: Imaginal exposure
ImRs: Imagery rescripting
ITQ: International Trauma Questionnaire
LEC-5: Life Events Checklist for DSM-5
MHQoL-7D: Mental Health Quality of Life seven-dimensional Questionnaire
PCL-5: PTSD Checklist for DSM-5
PDF: Personality Data Form
PE: Prolonged exposure
PFQ2: Personal Feelings Questionnaire 2
PTCI: Posttraumatic Cognitions Inventory
PTSD: Posttraumatic stress disorder
PTQ: Perseverative Thinking Questionnaire
RCT: Randomized clinical trial
SECS: Self-Expression and Control Scale
SCID-5-CV: Structural Clinical Interview for DSM-5-Clinician Version
SCID-5-PD: Structural Clinical Interview for DSM-5 Personality Disorders
SCID-5-RV: Structural Clinical Interview for DSM-5-Research Version
SCID-I: Structural Clinical Interview for DSM-IV Axis I disorders
SCID-II: Structural Clinical Interview for DSM-IV Axis II disorders
SCL-90-R: Symptom Checklist-90-Revised
SMI: Schema Mode Inventory
SPIRIT: Standard Protocol Items: Recommendations for Interventional Trials
TF-CBT: Trauma-focused cognitive behavior therapy
TVIC: Gordon Test of Visual Imagery Control
URICA: University of Rhode Island Change Assessment
VAS: Visual analog scale
WHODAS 2.0: World Health Organization Disability Assessment Schedule 2.0

## References

[1] American Psychiatric Association (2013). Diagnostic and statistical manual of mental disorders.

[2] Bisson JI, Roberts NP, Andrew M, Cooper R, Lewis C. Psychological therapies for chronic post-traumatic stress disorder (PTSD) in adults. Cochrane Database Syst Rev. 2013;12:CD003388. 10.1002/14651858.CD003388.pub4. 10.1002/14651858.CD003388.pub4 PMC6991463 24338345

[3] Bisson JI, Berliner L, Cloitre M, Forbes D, Jensen TK, Lewis C, et al. The international society for traumatic stress studies new guidelines for the prevention and treatment of posttraumatic stress disorder: Methodology and development process. J Trauma Stress. 2019;32(4):475–83. 10.1002/jts.22421. 10.1002/jts.22421 31283056

[4] Cloitre M, Courtois CA, Charuvastra A, Carapezza R, Stolbach BC, Green BL (2011). Treatment of complex PTSD: results of the ISTSS expert clinician survey on best practices. J Trauma Stress.

[5] Dorrepaal E, Thomaes K, Hoogendoorn AW, Veltman DJ, Draijer N, van Balkom AJ (2014). Evidence-based treatment for adult women with child abuse-related complex PTSD: a quantitative review. Eur J Psychotraumatol.

[6] De Jongh A, Resick PA, Zoellner LA, Van Minnen A, Lee CW, Monson CM (2016). Critical analysis of the current treatment guidelines for complex PTSD in adults. Depress Anxiety.

[7] Ehring T, Welboren R, Morina N, Wicherts JM, Freitag J, Emmelkamp PM (2014). Meta-analysis of psychological treatments for posttraumatic stress disorder in adult survivors of childhood abuse. Clin Psychol Rev.

[8] Herman JL, Complex PTSD (1992). A syndrome in survivors of prolonged and repeated trauma. J Trauma Stress.

[9] Messman-Moore TL, Bhuptani PH (2017). A review of the long-term impact of child maltreatment on posttraumatic stress disorder and its comorbidities: an emotion dysregulation perspective. Clin Psychol Sci Pract.

[10] Spinazzola J, Blaustein M, Van Der Kolk BA (2005). Posttraumatic stress disorder treatment outcome research: the study of unrepresentative samples?. J Trauma Stress.

[11] Boterhoven de Haan KL, Lee CW, Fassbinder E, van Es SM, Meewisse M, Menninga S (2020). Imagery rescripting and eye movement desensitisation and reprocessing as treatment for adults with post-traumatic stress disorder from childhood trauma: randomised clinical trial. Br J Psychiatry.

[12] Arntz A (2012). Imagery rescripting as a therapeutic technique: review of clinical trials, basic studies, and research agenda. J Exp Psychopathol.

[13] Arntz A, Weertman A (1999). Treatment of childhood memories: theory and practice. Behav Res Ther.

[14] Shapiro F, Forrest MS (2001). EMDR: eye movement desensitization and reprocessing.

[15] Morina N, Lancee J, Arntz A (2017). Imagery rescripting as a clinical intervention for aversive memories: a meta-analysis. J Behav Ther Exp Psychiatry.

[16] Raabe S, Ehring T, Marquenie L, Olff M, Kindt M (2015). Imagery rescripting as stand-alone treatment for posttraumatic stress disorder related to childhood abuse. J Behav Ther Exp Psychiatry.

[17] Gutner CA, Suvak MK, Sloan DM, Resick PA (2016). Does timing matter? Examining the impact of session timing on outcome. J Consult Clin Psychol.

[18] Ehlers A, Clark DM, Hackmann A, Grey N, Liness S, Wild J, et al. Intensive cognitive therapy for PTSD: a feasibility study. Behav Cogn Psychother. 2010;38(4):383–98. 10.1017/S1352465810000214. 10.1017/S1352465810000214 PMC2893530 20573292

[19] Ehlers A, Hackmann A, Grey N, Wild J, Liness S, Albert I, et al. A randomized controlled trial of 7-day intensive and standard weekly cognitive therapy for PTSD and emotion-focused supportive therapy. Am J Psychiatry. 2014;171(3):294–304. 10.1176/appi.ajp.2013.13040552. 10.1176/appi.ajp.2013.13040552 PMC4082238 24480899

[20] Hurley EC (2018). Effective treatment of veterans with PTSD: comparison between intensive daily and weekly EMDR approaches. Front Psychol.

[21] Murray H, El-Leithy S, Billings J (2017). Intensive cognitive therapy for post-traumatic stress disorder in routine clinical practice: a matched comparison audit. Br J Clin Psychol.

[22] Cuijpers P, Huibers M, Ebert DD, Koole SL, Andersson G (2013). How much psychotherapy is needed to treat depression? A metaregression analysis. J Affect Disord.

[23] Erekson DM, Lambert MJ, Eggett DL (2015). The relationship between session frequency and psychotherapy outcome in a naturalistic setting. J Consult Clin Psychol.

[24] Tiemens B, Kloos M, Spijker J, Ingenhoven T, Kampman M, Hendriks G (2019). Lower versus higher frequency of sessions in starting outpatient mental health care and the risk of a chronic course; a naturalistic cohort study. BMC Psychiatry.

[25] Bruijniks SJ, Lemmens LH, Hollon SD, Peeters FP, Cuijpers P, Arntz A (2020). The effects of once-versus twice-weekly sessions on psychotherapy outcomes in depressed patients. Br J Psychiatry.

[26] Arntz A, Lee CW, Ehring T, Nexhmedin M, Fassbinder E, Walton CJ, et al. The effect of session frequency on treatment of complex PTSD in adults by Imagery Rescripting (ImRs) and Eye Movement Desensitization and Reprocessing (EMDR). Study protocol. 2018.

[27] Bruijniks SJ, Bosmans J, Peeters FP, Hollon SD, van Oppen P, van den Boogaard M (2015). Frequency and change mechanisms of psychotherapy among depressed patients: study protocol for a multicenter randomized trial comparing twice-weekly versus once-weekly sessions of CBT and IPT. BMC Psychiatry.

[28] McLean CP, Foa EB (2011). Prolonged exposure therapy for post-traumatic stress disorder: a review of evidence and dissemination. Expert Rev Neurother.

[29] Rogers S, Silver SM (2002). Is EMDR an exposure therapy? A review of trauma protocols. J Clin Psychol.

[30] Henn FA, Vollmayr B (2004). Neurogenesis and depression: etiology or epiphenomenon?. Biol Psychiatry.

[31] Cloitre M, Chase Stovall-McClough K, Miranda R, Chemtob CM (2004). Therapeutic alliance, negative mood regulation, and treatment outcome in child abuse-related posttraumatic stress disorder. J Consult Clin Psychol.

[32] Hoffart A, Øktedalen T, Langkaas TF, Wampold BE (2013). Alliance and outcome in varying imagery procedures for PTSD: A study of within-person processes. J Couns Psychol.

[33] McLaughlin AA, Keller SM, Feeny NC, Youngstrom EA, Zoellner LA (2014). Patterns of therapeutic alliance: rupture–repair episodes in prolonged exposure for posttraumatic stress disorder. J Consult Clin Psychol.

[34] Boterhoven de Haan KL, Lee CW, Correia H, Menninga S, Fassbinder E, Köehne S, et al. Patient and Therapist Perspectives on Treatment for Adults with PTSD from Childhood Trauma. J Clin Med. 2021;10(5):954. 10.3390/jcm10050954. 10.3390/jcm10050954 PMC7957589 33804440

[35] Hendriks L, de Kleine RA, Broekman TG, Hendriks GJ, van Minnen A. Intensive prolonged exposure therapy for chronic PTSD patients following multiple trauma and multiple treatment attempts. Eur J Psychotraumatol. 2018;9(1). 10.1080/20008198.2018.1425574. 10.1080/20008198.2018.1425574 PMC5795659 29410776

[36] Oprel D, Hoeboer CM, Schoorl M, De Kleine RA, Wigard IG, Cloitre M (2018). Improving treatment for patients with childhood abuse related posttraumatic stress disorder (IMPACT study): protocol for a multicenter randomized trial comparing prolonged exposure with intensified prolonged exposure and phase-based treatment. BMC Psychiatry.

[37] Menninga S, Van Es SM, Boterhoven De Haan KL, Lee CW, Fassbinder E, Koehne S, et al. Patients’ perspective on the effective working mechanisms in ImRs and EMDR treating childhood-trauma-related PTSD: a qualitative study. Eur J Psychotraumatol. 2019;10(sup1):13. 10.1080/20008198.2019.1613834.

[38] Boterhoven de Haan KL, Lee CW, Fassbinder E, Voncken MJ, Meewisse M, Van Es SM, et al. Imagery rescripting and eye movement desensitisation and reprocessing for treatment of adults with childhood trauma-related post-traumatic stress disorder: IREM study design. BMC Psychiatry. 2017;17(1):165. 10.1186/s12888-017-1330-2. 10.1186/s12888-017-1330-2 PMC5418842 28472933

[39] Chan A, Tetzlaff JM, Gøtzsche PC, Altman DG, Mann H, Berlin JA (2013). SPIRIT 2013 explanation and elaboration: guidance for protocols of clinical trials. BMJ.

[40] Shapiro F (1989). Eye movement desensitization: a new treatment for post-traumatic stress disorder. J Behav Ther Exp Psychiatry.

[41] Cooper RZ, Smith AD, Lewis D, Lee CW, Leeds AM (2019). Developing the interrater reliability of the modified EMDR fidelity checklist. J EMDR Pract Res.

[42] Raabe S (2016). Imagery rescripting (ImRs) therapist adherence and competence protocol.

[43] First MB, Williams JB, Karg RS, Spitzer RL (2016). Structured clinical interview for DSM-5 Disorders, Clinician Version (SCID-5-CV).

[44] First MB, Williams J, Karg RS, Spitzer RL (2015). Structured clinical interview for DSM-5—Research Version (SCID-5 for DSM-5, Research Version; SCID-5-RV).

[45] First MB, Williams J, Benjamin LS, Spitzer RL (2015). User’s guide for the SCID-5-PD (Structured Clinical Interview for DSM-5 Personality Disorder).

[46] First MB, Gibbon M, Spitzer RL, Benjamin LS, Williams JB (1997). Structured clinical interview for DSM-IV Axis II personality disorders (SCID-II).

[47] Wittchen H, Zaudig M, Fydrich T (1997). Strukturiertes Klinisches Interview für DSM-IV, Achse I (SKID-I).

[48] Lobbestael J, Leurgans M, Arntz A (2011). Inter-rater reliability of the Structured Clinical Interview for DSM-IV Axis I disorders (SCID I) and Axis II disorders (SCID II). Clin Psychol Psychother.

[49] Maffei C, Fossati A, Agostoni I, Barraco A, Bagnato M, Deborah D, et al. Interrater reliability and internal consistency of the structured clinical interview for DSM-IV axis II personality disorders (SCID-II), version 2.0. J Personal Disord. 1997;11(3):279–84. 10.1521/pedi.1997.11.3.279. 10.1521/pedi.1997.11.3.279 9348491

[50] Zanarini MC, Skodol AE, Bender D, Dolan R, Sanislow C, Schaefer E, et al. The collaborative longitudinal personality disorders study: reliability of axis I and II diagnoses. J Personal Disord. 2000;14(4):291–9. 10.1521/pedi.2000.14.4.291. 10.1521/pedi.2000.14.4.291 11213787

[51] Osório FL, Loureiro SR, Hallak JEC, Machado-de-Sousa JP, Ushirohira JM, Baes CV (2019). Clinical validity and intrarater and test–retest reliability of the Structured Clinical Interview for DSM-5–Clinician Version (SCID-5-CV). Psychiatry Clin Neurosci.

[52] Somma A, Borroni S, Maffei C, Besson E, Garbini A, Granozio S (2017). Inter-rater reliability of the Italian translation of the structured clinical interview for DSM-5 personality disorders (SCID-5-PD): a study on consecutively admitted clinical adult participants. J Psychopathol.

[53] Weathers FW, Blake DD, Schnurr PP, Kaloupek DG, Marx BP, Keane TM (2013). The life events checklist for DSM-5 (LEC-5).

[54] Weathers FW, Blake DD, Schnurr PP, Kaloupek DG, Marx BP, Keane TM (2013). The clinician-administered PTSD scale for DSM-5 (CAPS-5).

[55] Boeschoten MA, Van der Aa N, Bakker A, Ter Heide FJJ, Hoofwijk MC, Jongedijk RA, et al. Development and evaluation of the Dutch clinician-administered PTSD scale for DSM-5 (CAPS-5). Eur J Psychotraumatol. 2018;9(1). 10.1080/20008198.2018.1546085. 10.1080/20008198.2018.1546085 PMC6263102 30510643

[56] Müller-Engelmann M, Schnyder U, Dittmann C, Priebe K, Bohus M, Thome J, et al. Psychometric properties and factor structure of the German version of the clinician-administered PTSD scale for DSM-5. Assessment. 2018;27(6):1128–38 1073191118774840. 10.1177/1073191118774840 10.1177/1073191118774840 29766744

[57] Weathers FW, Bovin MJ, Lee DJ, Sloan DM, Schnurr PP, Kaloupek DG, et al. The Clinician-Administered PTSD Scale for DSM-5 (CAPS-5): development and initial psychometric evaluation in military veterans. Psychol Assess. 2018;30(3):383–95. 10.1037/pas0000486. 10.1037/pas0000486 PMC5805662 28493729

[58] Weathers FW, Litz BT, Keane TM, Palmieri PA, Marx BP, Schnurr PP (2013). The PTSD Checklist for DSM-5 (PCL-5).

[59] Blevins CA, Weathers FW, Davis MT, Witte TK, Domino JL (2015). The posttraumatic stress disorder checklist for DSM-5 (PCL-5): Development and initial psychometric evaluation. J Trauma Stress.

[60] Bovin MJ, Marx BP, Weathers FW, Gallagher MW, Rodriguez P, Schnurr PP, et al. Psychometric properties of the PTSD checklist for diagnostic and statistical manual of mental disorders–fifth edition (PCL-5) in veterans. Psychol Assess. 2016;28(11):1379–91. 10.1037/pas0000254. 10.1037/pas0000254 26653052

[61] Wortmann JH, Jordan AH, Weathers FW, Resick PA, Dondanville KA, Hall-Clark B, et al. Psychometric analysis of the PTSD Checklist-5 (PCL-5) among treatment-seeking military service members. Psychol Assess. 2016;28(11):1392–403. 10.1037/pas0000260. 10.1037/pas0000260 26751087

[62] Arntz A, Tiesema M, Kindt M (2007). Treatment of PTSD: a comparison of imaginal exposure with and without imagery rescripting. J Behav Ther Exp Psychiatry.

[63] Foa EB, Ehlers A, Clark DM, Tolin DF, Orsillo SM (1999). The posttraumatic cognitions inventory (PTCI): development and validation. Psychol Assess.

[64] Beck AT, Steer RA, Brown G (1996). Beck depression inventory–II.

[65] Dozois DJ, Dobson KS, Ahnberg JL (1998). A psychometric evaluation of the Beck Depression Inventory–II. Psychol Assess.

[66] Harder DH, Zalma A (1990). Two promising shame and guilt scales: a construct validity comparison. J Pers Assess.

[67] Hoblitzelle W. Attempts to measure and differentiate shame and guilt: The relationship between shame and depression. In: Lewis HB, editor. The role of shame in symptom formation. Hillsdale: Erlbaum; 1987. p 207–235.

[68] Arntz A, Ehring T, Nexhmedin M, Lee CW (2018). The guilt and shame questionnaire.

[69] van Elderen T, Maes S, Komproe I, van der Kamp L (1997). The development of an anger expression and control scale. Br J Health Psychol.

[70] Derogatis LR (1977). The SCL-90 Manual I: Scoring, administration and procedures for the SCL-90.

[71] Derogatis LR, Unger R. Symptom checklist-90-revised. In: Weiner IB, Craighead WE, editors. The Corsini encyclopedia of psychology. New York: Wiley; 2010. 10.1002/9780470479216.corpsy0970.

[72] van Elderen T, Verkes R, Arkesteijn J, Komproe I (1996). Psychometric characteristics of the self-expression and control scale in a sample of recurrent suicide attempters. Pers individ Differ.

[73] Cavalcanti JG, Moura GBd, Pimentel CE. (2019). Psychometric parameters of the subscale of hostility from the Symptom Checklist 90 (SCL-90). Psico-USF.

[74] Derogatis LR, Rickels K, Rock AF. The SCL-90 and the MMPI: A step in the validation of a new self-report scale. Br J Psychiatry. 1976;128:280–9. 10.1192/bjp.128.3.280. 10.1192/bjp.128.3.280 1252693

[75] Abdel-Khalek AM (2006). Measuring happiness with a single-item scale. Soc Behav Pers Int J.

[76] Kleim B, Gonzalo D, Ehlers A (2011). The Depressive Attributions Questionnaire (DAQ): Development of a short self-report measure of depressogenic attributions. J Psychopathol Behav Assess.

[77] Schierholz A, Krüger A, Barenbrügge J, Ehring T (2016). What mediates the link between childhood maltreatment and depression? The role of emotion dysregulation, attachment, and attributional style. Eur J Psychotraumatol.

[78] Waller N, Putnam FW, Carlson EB (1996). Types of dissociation and dissociative types: a taxometric analysis of dissociative experiences. Psychol Methods.

[79] Bernstein EM, Putnam FW (1986). Development, reliability, and validity of a dissociation scale. J Nerv Ment Dis.

[80] Spitzer C, Freyberger H, Brähler E, Beutel ME, Stieglitz R (2015). Psychometric evaluation of the Dissociative Experiences Scale-Taxon (DES-T). Psychother Psychosom Med Psychol.

[81] Herdman M, Gudex C, Lloyd A, Janssen MF, Kind P, Parkin D, et al. Development and preliminary testing of the new five-level version of EQ-5D (EQ-5D-5L). Quality of life research. 2011;20(10):1727–36. 10.1007/s11136-011-9903-x. 10.1007/s11136-011-9903-x PMC3220807 21479777

[82] van Krugten F, van Busschbach JJ, Versteegh MM, Hakkaart-van Roijen L, Brouwer W. The Mental Health Quality of Life Seven-Dimensional Questionnaire (MHQoL-7D): Development and first psychometric evaluation of a new measure to assess quality of life in people with mental health problems. 2019 World Congress on Health Economics: iHEA; 2019. 10.1007/s11136-021-02935-w PMC8847188 34241821

[83] Janssen MF, Pickard AS, Golicki D, Gudex C, Niewada M, Scalone L, et al. Measurement properties of the EQ-5D-5L compared to the EQ-5D-3L across eight patient groups: a multi-country study. Quality of Life Research. 2013;22(7):1717–27. 10.1007/s11136-012-0322-4. 10.1007/s11136-012-0322-4 PMC3764313 23184421

[84] Cloitre M, Shevlin M, Brewin CR, Bisson JI, Roberts NP, Maercker A, et al. The International Trauma Questionnaire: development of a self-report measure of ICD-11 PTSD and complex PTSD. Acta Psychiatr Scand. 2018;138(6):536–46. 10.1111/acps.12956. 10.1111/acps.12956 30178492

[85] World Health Organization. ICD-11 for mortality and morbidity statistics. 2018; https://icd.who.int/browse11/l-m/en.

[86] Hyland P, Shevlin M, Brewin CR, Cloitre M, Downes AJ, Jumbe S, et al. Validation of post-traumatic stress disorder (PTSD) and complex PTSD using the International Trauma Questionnaire. Acta Psychiatr Scand. 2017;136(3):313–22. 10.1111/acps.12771. 10.1111/acps.12771 28696531

[87] Üstün TB, Kostanjsek N, Chatterji S, Rehm J (2010). Measuring health and disability: Manual for WHO disability assessment schedule WHODAS 2.0.

[88] Engelhard IM, van den Hout MA, Smeets MA (2011). Taxing working memory reduces vividness and emotional intensity of images about the Queen’s Day tragedy. J Behav Ther Exp Psychiatry.

[89] Lee SW, Kwon J (2013). The efficacy of imagery rescripting (IR) for social phobia: a randomized controlled trial. J Behav Ther Exp Psychiatry.

[90] van den Hout MA, Engelhard IM (2012). How does EMDR work?. J Exp Psychopathol.

[91] Wild J, Hackmann A, Clark DM (2007). When the present visits the past: updating traumatic memories in social phobia. J Behav Ther Exp Psychiatry.

[92] Bernstein DP, Stein JA, Newcomb MD, Walker E, Pogge D, Ahluvalia T, et al. Development and validation of a brief screening version of the Childhood Trauma Questionnaire. Child Abuse Negl. 2003;27(2):169–90. 10.1016/S0145-2134(02)00541-0. 10.1016/s0145-2134(02)00541-0 12615092

[93] Karos K, Niederstrasser N, Abidi L, Bernstein DP, Bader K (2014). Factor structure, reliability, and known groups validity of the German version of the Childhood Trauma Questionnaire (Short-form) in Swiss patients and nonpatients. J Child Sex Abuse.

[94] Thombs BD, Bernstein DP, Lobbestael J, Arntz A (2009). A validation study of the Dutch Childhood Trauma Questionnaire-Short Form: factor structure, reliability, and known-groups validity. Child Abuse Negl.

[95] Gámez W, Chmielewski M, Kotov R, Ruggero C, Suzuki N, Watson D (2014). The Brief Experiential Avoidance Questionnaire: development and initial validation. Psychol Assess.

[96] DiClemente CC, Bellino LE, Neavins TM (1999). Motivation for change and alcoholism treatment. Alcohol Res Health.

[97] DiClemente CC, Hughes SO (1990). Stages of change profiles in outpatient alcoholism treatment. J Subst Abuse.

[98] McConnaughy EA, Prochaska JO, Velicer WF (1983). Stages of change in psychotherapy: Measurement and sample profiles. Psychother Theory Res Pract.

[99] Carbonari JP, DiClemente CC, Zweben A. A readiness to change scale: its development, validation, and usefulness. The annual meeting of the Association for Advancement of Behavior Therapy, San Diego, CA. 1994.

[100] Clark DM (2011). Implementing NICE guidelines for the psychological treatment of depression and anxiety disorders: the IAPT experience. Int Rev Psychiatry.

[101] John OP, Donahue EM, Kentle RL (1991). The Big Five Inventory—Versions 4a and 54.

[102] John OP, Donahue EM, Kentle RL (1998). The big five inventory: studies of reliability and validity.

[103] Luteijn F, Van der Ploeg F (1983). Handleiding bij de GIT [Manual for the GIT].

[104] Luteijn F, Barelds DPH. GIT2: Groninger Intelligentie Test 2: Harcourt Test Publishers; 2004.

[105] Bamelis LL, Renner F, Heidkamp D, Arntz A (2011). Extended schema mode conceptualizations for specific personality disorders: an empirical study. J Personal Disord.

[106] Young JE, Arntz A, Atkinson T, Lobbestael J, Weishaar ME, Van Vreeswijk MF (2007). The schema mode inventory.

[107] Lobbestael J, van Vreeswijk M, Spinhoven P, Schouten E, Arntz A (2010). Reliability and validity of the short Schema Mode Inventory (SMI). Behav Cogn Psychother.

[108] Richardson A (1969). Mental imagery.

[109] Pérez-Fabello MJ, Campos A (2004). Factor structure and internal consistency of the Spanish version of the Gordon Test of Visual Imagery Control. Psychol Rep.

[110] Westcott TB, Rosenstock E (1976). Reliability of two measures of imagery. Percept Mot Skills.

[111] White K, Sheehan PW, Ashton R. Imagery assessment: a survey of self-report measures. J Ment Imagery. 1977;1(1):145–169.

[112] Fraley RC, Heffernan ME, Vicary AM, Brumbaugh CC (2011). The experiences in close relationships—Relationship Structures Questionnaire: a method for assessing attachment orientations across relationships. Psychol Assess.

[113] Fraley RC, Waller NG, Brennan KA (2000). An item response theory analysis of self-report measures of adult attachment. J Pers Soc Psychol.

[114] Ellis A (1968). Personality data form.

[115] Shorkey CT, Sutton-Simon K (1983). Personality data form: initial reliability and validity. Psychol Rep.

[116] Ehring T, Zetsche U, Weidacker K, Wahl K, Schönfeld S, Ehlers A (2011). The Perseverative Thinking Questionnaire (PTQ): validation of a content-independent measure of repetitive negative thinking. J Behav Ther Exp Psychiatry.

[117] Ehring T, Raes F, Weidacker K, Emmelkamp PM (2012). Validation of the Dutch version of the Perseverative Thinking Questionnaire (PTQ-NL). Eur J Psychol Assess.

[118] Granger CW (1969). Investigating causal relations by econometric models and cross-spectral methods. Econometrica: journal of the Econometric Society.

[119] Keefe JR, Wiltsey Stirman S, Cohen ZD, DeRubeis RJ, Smith BN, Resick PA (2018). In rape trauma PTSD, patient characteristics indicate which trauma-focused treatment they are most likely to complete. Depress Anxiety.

[120] Zilcha-Mano S, Keefe JR, Chui H, Rubin A, Barrett MS, Barber JP (2016). Reducing dropout in treatment for depression: translating dropout predictors into individualized treatment recommendations. J Clin Psychiatry.

[121] Fournier JC, DeRubeis RJ, Shelton RC, Hollon SD, Amsterdam JD, Gallop R (2009). Prediction of response to medication and cognitive therapy in the treatment of moderate to severe depression. J Consult Clin Psychol.

[122] Epskamp S, Borsboom D, Fried EI (2018). Estimating psychological networks and their accuracy: A tutorial paper. Behavior Research Methods.

[123] Epskamp S, Waldorp LJ, Mõttus R, Borsboom D (2018). The Gaussian graphical model in cross-sectional and time-series data. Multivariate Behav Res.

[124] Leer A, Engelhard IM, Van Den Hout MA (2014). How eye movements in EMDR work: changes in memory vividness and emotionality. J Behav Ther Exp Psychiatry.

[125] Reimer SG, Moscovitch DA (2015). The impact of imagery rescripting on memory appraisals and core beliefs in social anxiety disorder. Behav Res Ther.

[126] van den Hout MA, Bartelski N, Engelhard IM (2013). On EMDR: eye movements during retrieval reduce subjective vividness and objective memory accessibility during future recall. Cogn Emot.

[127] Newton-Howes G, Tyrer P, Johnson T (2006). Personality disorder and the outcome of depression: meta-analysis of published studies. Br J Psychiatry.

[128] Weertman A, Arntz A, Schouten E, Dreessen L (2005). Influences of beliefs and personality disorders on treatment outcome in anxiety patients. J Consult Clin Psychol.

[129] Schottenbauer MA, Glass CR, Arnkoff DB, Tendick V, Gray SH (2008). Nonresponse and dropout rates in outcome studies on PTSD: review and methodological considerations. Psychiatry.

[130] Cougle JR, Timpano KR, Sachs-Ericsson N, Keough ME, Riccardi CJ (2010). Examining the unique relationships between anxiety disorders and childhood physical and sexual abuse in the National Comorbidity Survey-Replication. Psychiatry Res.

[131] Cecil CA, Viding E, Fearon P, Glaser D, McCrory EJ (2017). Disentangling the mental health impact of childhood abuse and neglect. Child Abuse Negl.

[132] Evren C, Umut G, Bozkurt M, Evren B, Agachanli R (2016). Mediating role of childhood emotional abuse on the relationship between severity of ADHD and PTSD symptoms in a sample of male inpatients with alcohol use disorder. Psychiatry Res.

[133] Grassi-Oliveira R, Stein LM (2008). Childhood maltreatment associated with PTSD and emotional distress in low-income adults: the burden of neglect. Child Abuse Negl.

[134] Schumm JA, Dickstein BD, Walter KH, Owens GP, Chard KM (2015). Changes in posttraumatic cognitions predict changes in posttraumatic stress disorder symptoms during cognitive processing therapy. J Consult Clin Psychol.

[135] Talamini LM, Gorree E (2012). Aging memories: differential decay of episodic memory components. Learning & Memory.

[136] Tunney RJ (2010). Do changes in the subjective experience of recognition over time suggest independent processes?. Br J Math Stat Psychol.

[137] de Oliveira Alvares L, Einarsson EÖ, Santana F, Crestani AP, Haubrich J, Cassini LF, et al. Periodically reactivated context memory retains its precision and dependence on the hippocampus. Hippocampus. 2012;22(5):1092–5. 10.1002/hipo.20983. 10.1002/hipo.20983 22120981

[138] Overmier JB (2002). On learned helplessness. Integr Physiol Behav Sci.

[139] Eğeci İS, Özgün S (2019). Randomized Controlled Trial: EMDR Early Intervention With and Without Eye Movements for Learned Helplessness State. J EMDR Pract Res.

[140] Barawi KS, Lewis C, Simon N, Bisson JI (2020). A systematic review of factors associated with outcome of psychological treatments for post-traumatic stress disorder. Eur J Psychotraumatol.

[141] Borsboom D (2017). A network theory of mental disorders. World Psychiatry.

[142] Birkeland MS, Greene T, Spiller TR (2020). The network approach to posttraumatic stress disorder: A systematic review. Eur J Psychotraumatol.

[143] Segal A, Wald I, Lubin G, Fruchter E, Ginat K, Ben Yehuda A, et al. Changes in the dynamic network structure of PTSD symptoms pre-to-post combat. Psychol Med. 2019;50(5):746–753. 10.1017/s0033291719000539. 10.1017/S0033291719000539 PMC7690054 30919787

[144] Shalom JG, Aderka IM (2020). A meta-analysis of sudden gains in psychotherapy: outcome and moderators. Clin Psychol Rev.

[145] Qualtrics (2019). Qualtrics.

[146] Finley EP, Garcia HA, Ketchum NS, McGeary DD, McGeary CA, Stirman SW (2015). Utilization of evidence-based psychotherapies in Veterans Affairs posttraumatic stress disorder outpatient clinics. Psychol Serv.

[147] Kazdin AE, Nock MK (2003). Delineating mechanisms of change in child and adolescent therapy: Methodological issues and research recommendations. J Child Psychol Psychiatry.

[148] Huibers M (2015). Voorbij het oordeel van de dodo. Tijdschrift voor psychotherapie.

[149] Ronconi JM, Shiner B, Watts BV. Inclusion and exclusion criteria in randomized controlled trials of psychotherapy for PTSD. J Psychiatr Pract. 2014;20(1):25–37. 10.1097/01.pra.0000442936.23457.5b. 10.1097/01.pra.0000442936.23457.5b 24419308

[150] Leykin Y, DeRubeis RJ (2009). Allegiance in psychotherapy outcome research: separating association from bias. Clin Psychol Sci Pract.

[151] Westen D, Novotny CM, Thompson-Brenner H (2004). The empirical status of empirically supported psychotherapies: assumptions, findings, and reporting in controlled clinical trials. Psychol Bull.

[152] Lemmens LH, Galindo-Garre F, Arntz A, Peeters F, Hollon SD, DeRubeis RJ (2017). Exploring mechanisms of change in cognitive therapy and interpersonal psychotherapy for adult depression. Behav Res Ther.

