# Supplementary material for: The effect of twice-weekly versus once-weekly sessions of either imagery rescripting or eye movement desensitization and reprocessing for adults with PTSD from childhood trauma (IREM-Freq): a study protocol for an international randomized clinical trial
Source: Trials. 2021 Nov 27;22:848. doi: 10.1186/s13063-021-05712-9 (PMC8626728; doi:10.1186/s13063-021-05712-9)
Supplement: Supplementary file 1 — Additional file 1. [file 13063_2021_5712_MOESM1_ESM.docx]

**Additional file 1**

**Appendix A. SPIRIT 2013 Checklist**

SPIRIT 2013 Checklist: Recommended items to address in a clinical trial protocol and related documents*

| Section/item | ItemNo | Description | Addressed on page number |
| --- | --- | --- | --- |
| **Administrative information** | | |  |
| Title | 1 | Descriptive title identifying the study design, population, interventions, and, if applicable, trial acronym | 1, 6 |
| Trial registration | 2a | Trial identifier and registry name. If not yet registered, name of intended registry | 6, 12 |
|  | 2b | All items from the World Health Organization Trial Registration Data Set | 12 |
| Protocol version | 3 | Date and version identifier | 6, 39 |
| Funding | 4 | Sources and types of financial, material, and other support | 6, 42 |
| Roles and responsibilities | 5a | Names, affiliations, and roles of protocol contributors | 42-44 |
|  | 5b | Name and contact information for the trial sponsor | 8 |
|  | 5c | Role of study sponsor and funders, if any, in study design; collection, management, analysis, and interpretation of data; writing of the report; and the decision to submit the report for publication, including whether they will have ultimate authority over any of these activities | 8,42 |
|  | 5d | Composition, roles, and responsibilities of the coordinating centre, steering committee, endpoint adjudication committee, data management team, and other individuals or groups overseeing the trial, if applicable (see Item 21a for data monitoring committee) | 34, 35, Appendix J |
| Introduction |  |  |  |
| Background and rationale | 6a | Description of research question and justification for undertaking the trial, including summary of relevant studies (published and unpublished) examining benefits and harms for each intervention | 8-12 |
|  | 6b | Explanation for choice of comparators | 8, 9 |
| Objectives | 7 | Specific objectives or hypotheses | 11, 12 |
| Trial design | 8 | Description of trial design including type of trial (eg, parallel group, crossover, factorial, single group), allocation ratio, and framework (eg, superiority, equivalence, noninferiority, exploratory) | 12 |

| Methods: Participants, interventions, and outcomes | | |  |
| --- | --- | --- | --- |
| Study setting | 9 | Description of study settings (eg, community clinic, academic hospital) and list of countries where data will be collected. Reference to where list of study sites can be obtained | 13 |
| Eligibility criteria | 10 | Inclusion and exclusion criteria for participants. If applicable, eligibility criteria for study centres and individuals who will perform the interventions (eg, surgeons, psychotherapists) | 13, 14 |
| Interventions | 11a | Interventions for each group with sufficient detail to allow replication, including how and when they will be administered | 17-19 |
|  | 11b | Criteria for discontinuing or modifying allocated interventions for a given trial participant (eg, drug dose change in response to harms, participant request, or improving/worsening disease) | 17, 20 |
|  | 11c | Strategies to improve adherence to intervention protocols, and any procedures for monitoring adherence (eg, drug tablet return, laboratory tests) | 16, 20 |
|  | 11d | Relevant concomitant care and interventions that are permitted or prohibited during the trial | 20, 21 |
| Outcomes | 12 | Primary, secondary, and other outcomes, including the specific measurement variable (eg, systolic blood pressure), analysis metric (eg, change from baseline, final value, time to event), method of aggregation (eg, median, proportion), and time point for each outcome. Explanation of the clinical relevance of chosen efficacy and harm outcomes is strongly recommended | 21-29 |
| Participant timeline | 13 | Time schedule of enrolment, interventions (including any run-ins and washouts), assessments, and visits for participants. A schematic diagram is highly recommended (see Figure) | 15-17, Table 1 |
| Sample size | 14 | Estimated number of participants needed to achieve study objectives and how it was determined, including clinical and statistical assumptions supporting any sample size calculations | 14 |
| Recruitment | 15 | Strategies for achieving adequate participant enrolment to reach target sample size | 15 |
| **Methods: Assignment of interventions (for controlled trials)** | | |  |
| Allocation: |  |  |  |
| Sequence generation | 16a | Method of generating the allocation sequence (eg, computer-generated random numbers), and list of any factors for stratification. To reduce predictability of a random sequence, details of any planned restriction (eg, blocking) should be provided in a separate document that is unavailable to those who enrol participants or assign interventions | 17 |
| Allocation concealment mechanism | 16b | Mechanism of implementing the allocation sequence (eg, central telephone; sequentially numbered, opaque, sealed envelopes), describing any steps to conceal the sequence until interventions are assigned | 15-17 |
| Implementation | 16c | Who will generate the allocation sequence, who will enrol participants, and who will assign participants to interventions | 15-17 |
| Blinding (masking) | 17a | Who will be blinded after assignment to interventions (eg, trial participants, care providers, outcome assessors, data analysts), and how | 15, 16 |
|  | 17b | If blinded, circumstances under which unblinding is permissible, and procedure for revealing a participant’s allocated intervention during the trial | 16, 17 |
| **Methods: Data collection, management, and analysis** | | |  |
| Data collection methods | 18a | Plans for assessment and collection of outcome, baseline, and other trial data, including any related processes to promote data quality (eg, duplicate measurements, training of assessors) and a description of study instruments (eg, questionnaires, laboratory tests) along with their reliability and validity, if known. Reference to where data collection forms can be found, if not in the protocol | 21-29 |
|  | 18b | Plans to promote participant retention and complete follow-up, including list of any outcome data to be collected for participants who discontinue or deviate from intervention protocols | 16 |
| Data management | 19 | Plans for data entry, coding, security, and storage, including any related processes to promote data quality (eg, double data entry; range checks for data values). Reference to where details of data management procedures can be found, if not in the protocol | 35, 36 |
| Statistical methods | 20a | Statistical methods for analysing primary and secondary outcomes. Reference to where other details of the statistical analysis plan can be found, if not in the protocol | 29-31 |
|  | 20b | Methods for any additional analyses (eg, subgroup and adjusted analyses) | 29-31 |
|  | 20c | Definition of analysis population relating to protocol non-adherence (eg, as randomised analysis), and any statistical methods to handle missing data (eg, multiple imputation) | 30 |
| **Methods: Monitoring** | | |  |
| Data monitoring | 21a | Composition of data monitoring committee (DMC); summary of its role and reporting structure; statement of whether it is independent from the sponsor and competing interests; and reference to where further details about its charter can be found, if not in the protocol. Alternatively, an explanation of why a DMC is not needed | 35 |
|  | 21b | Description of any interim analyses and stopping guidelines, including who will have access to these interim results and make the final decision to terminate the trial | 30 |
| Harms | 22 | Plans for collecting, assessing, reporting, and managing solicited and spontaneously reported adverse events and other unintended effects of trial interventions or trial conduct | 35 |
| Auditing | 23 | Frequency and procedures for auditing trial conduct, if any, and whether the process will be independent from investigators and the sponsor | 35 |
| Ethics and dissemination | | |  |
| Research ethics approval | 24 | Plans for seeking research ethics committee/institutional review board (REC/IRB) approval | 42 |
| Protocol amendments | 25 | Plans for communicating important protocol modifications (eg, changes to eligibility criteria, outcomes, analyses) to relevant parties (eg, investigators, REC/IRBs, trial participants, trial registries, journals, regulators) | 12 |
| Consent or assent | 26a | Who will obtain informed consent or assent from potential trial participants or authorised surrogates, and how (see Item 32) | 15 |
|  | 26b | Additional consent provisions for collection and use of participant data and biological specimens in ancillary studies, if applicable | 16 |
| Confidentiality | 27 | How personal information about potential and enrolled participants will be collected, shared, and maintained in order to protect confidentiality before, during, and after the trial | 35, 36 |
| Declaration of interests | 28 | Financial and other competing interests for principal investigators for the overall trial and each study site | 42 |
| Access to data | 29 | Statement of who will have access to the final trial dataset, and disclosure of contractual agreements that limit such access for investigators | 36 |
| Ancillary and post-trial care | 30 | Provisions, if any, for ancillary and post-trial care, and for compensation to those who suffer harm from trial participation | 20 |
| Dissemination policy | 31a | Plans for investigators and sponsor to communicate trial results to participants, healthcare professionals, the public, and other relevant groups (eg, via publication, reporting in results databases, or other data sharing arrangements), including any publication restrictions | 35, 36 |
|  | 31b | Authorship eligibility guidelines and any intended use of professional writers | 36, 43 |
|  | 31c | Plans, if any, for granting public access to the full protocol, participant-level dataset, and statistical code | 36 |
| Appendices |  |  |  |
| Informed consent materials | 32 | Model consent form and other related documentation given to participants and authorised surrogates | Appendix B |
| Biological specimens | 33 | Plans for collection, laboratory evaluation, and storage of biological specimens for genetic or molecular analysis in the current trial and for future use in ancillary studies, if applicable | 16 |

*It is strongly recommended that this checklist be read in conjunction with the SPIRIT 2013 Explanation & Elaboration for important clarification on the items. Amendments to the protocol should be tracked and dated. The SPIRIT checklist is copyrighted by the SPIRIT Group under the Creative Commons “[Attribution-NonCommercial-NoDerivs 3.0 Unported](http://www.creativecommons.org/licenses/by-nc-nd/3.0/)” license.

**Appendix B. Informed consent**

I agree to participate in the above research project and give my consent freely.

I have read the Information Sheet provided and been given a full explanation of the purpose of this study, the procedures involved and of what is expected of me.

I understand that the project will be conducted as described in the Information Sheet, a copy of which I have retained.

I understand I can withdraw for the project at any time and do not have to give any reason for withdrawing.

I understand that I will be asked to undergo psychological treatment using either Imagery Rescripting or Eye Movement Desensitization and Reprocessing. This will require me to:

Attend a maximum of 12, 90-minute sessions either once or twice a week of either Imagery Rescripting or Eye Movement Desensitization and Reprocessing.

Attend sessions either once a week for 12 weeks or twice a week for six weeks.

Complete self-report questionnaires and be interviewed at different points including: prior to starting treatment, after treatment has finished, 8 weeks after treatment finished, and at a 1-year follow up.

I understand that I may be asked to attend an additional interview after my treatment has finished.

I understand I am free to withdraw from the study at any time prior to the conclusion of the treatment, before the end of the 12 sessions, without needing to give any reason.

I understand that my personal information will be kept confidential to the researchers and appropriate Sexual Assault Resource Centre staff unless required otherwise by law.

I understand that my name and identity will be stored separately from the data. All data provided by me will be analysed anonymously using code numbers.

I understand that this data may be used for future research to help better our understanding of the treatment of PTSD and of trauma memories.

Any data that is used in future research will not be able to identify me specifically.

I understand that my therapy sessions will be video-recorded for the purposes of ensuring that the therapist is providing the treatment consistently.

I have had the opportunity to have questions answered to my satisfaction.

I do/do not (circle appropriate) want a summary of the study posted to me when the study is complete (I acknowledge that this may take more than two years).

Print Name:

Signature: Date:

**Appendix C. Overview of syndromal disorders assessed with the extended SCID-5-CV**

| **Module** | **Content** | **Time Period** |
| --- | --- | --- |
| *Module A: Mood Episodes and Persistent Depressive Disorder* | Major Depressive Episode | Current (past month) and past |
|  | Manic Episode | Current (past month) and past |
|  | Hypomanic Episode | Current (past month) and past |
|  | Persistent Depressive Disorder | Current (past 2 years) |
| *Module B: Psychotic and Associated Symptoms* | Delusions | Lifetime |
|  | Hallucinations | Lifetime |
|  | Disorganized Speech and Behaviour | Lifetime |
|  | Catatonic Behaviour | Lifetime |
|  | Negative Symptoms | Lifetime |
| *Module C: Differential Diagnosis of Psychotic Disorders* | Schizophrenia | Current (past month) and past |
|  | Schizophreniform Disorder | Current (past month) and past |
|  | Schizoaffective Disorder | Current (past month) and past |
|  | Delusional Disorder | Current (past month) and past |
|  | Brief Psychotic Disorder | Current (past month) and past |
|  | Other Specified Psychotic Disorder | Current (past month) and past (remission) |
|  | Psychotic Disorder Due to AMC | Lifetime |
|  | Substance/Medication‐Induced Psychotic Disorder | Lifetime |
| *Module D: Differential Diagnosis of Mood Disorders* | Bipolar I Disorder | Current (past month) and past (remission) |
|  | Bipolar II Disorder | Current (past month) and past (remission) |
|  | Other Specified/Unspecified Bipolar Disorder | Current (past month) and past (remission) |
|  | Bipolar Disorder Due to AMC | Lifetime |
|  | Substance/Medication-Induced Bipolar Disorder | Lifetime |
|  | Major Depressive Disorder | Current (past month) and past (remission) |
|  | Other Specified/Unspecified Depressive Disorder | Current (past month) and past (remission) |
|  | Depressive Disorder Due to AMC | Lifetime |
|  | Substance/Medication-Induced Depressive Disorder | Lifetime |
| *Module E: Substance Use Disorders* | Alcohol Use Disorder | Current (past 12 months) |
|  | Sedative, Hypnotic, or Anxiolytic Use Disorder | Current (past 12 months) |
|  | Cannabis Use Disorder | Current (past 12 months) |
|  | Stimulant Use Disorder | Current (past 12 months) |
|  | Opioid Use Disorder | Current (past 12 months) |
|  | Phencyclidine Use Disorder | Current (past 12 months) |
|  | Other Hallucinogen Use Disorder | Current (past 12 months) |
|  | Inhalant Use Disorder | Current (past 12 months) |
|  | Sedative/Hypnotic/Anxiolytic Use Disorder | Current (past 12 months) |
|  | Other or Unknown Substance Use Disorder | Current (past 12 months) |
|  | Gambling Disorder | Current (past 12 months) |
| *Module F: Anxiety Disorders* | Panic Disorder | Current (past month) and past |
|  | Agoraphobia | Current (past 6 months) |
|  | Social Anxiety Disorder | Current (past 6 months) |
|  | Generalized Anxiety Disorder | Current (past 6 months) |
|  | Anxiety Disorder Due to AMC | Lifetime |
|  | Substance/Medication‐Induced Anxiety Disorder | Lifetime |
|  | Specific Phobia | Current (past 6 months) |

| **Module** | **Content** | **Time Period** |
| --- | --- | --- |
| *Module G: Obsessive‐*  *Compulsive and Related Disorders* | Obsessive‐Compulsive Disorder | Current (past month) |
|  | OC and Related Disorder Due to AMC | Lifetime |
|  | Substance/Medication‐Induced Obsessive‐Compulsive and Related Disorder | Lifetime |
|  | Body Dysmorphic Disorder | Current (past month) |
| *Module H: Posttraumatic Stress Disorder* | Posttraumatic Stress Disorder | Current (past month) and past |
| *Module I: Externalising Disorders* | Attention‐Deficit/Hyperactivity Disorder | Current (past 6 months) |
|  | Intermittent Explosive Disorder | Current (past 12 months) |
| *Module J: Feeding and Eating Disorders* | Anorexia Nervosa | Current (past 3 months) and past |
|  | Bulimia Nervosa | Current (past 3 months) |
|  | Binge‐Eating Disorder | Current (past 3 months) |
|  | Avoidant/Restrictive Food Intake Disorder | Current (past month) |
|  | Other Specified Feeding or Eating Disorder | Current |
| *Module K: Somatic Symptom and Related Disorders* | Somatic Symptom Disorder | Current (past 6 months) |
|  | Illness Anxiety Disorder | Current (past 6 months) |
| *Module L: Sleep Disorders* | Insomnia Disorder | Current (past 3 months) |
|  | Hypersomnolence Disorder | Current (past 3 months) |
|  | Nightmare disorder | Current (past month) |
| *Module M: Screening for other disorders* | Separation Anxiety Disorder | n/a |
|  | Hoarding Disorder | n/a |
|  | Trichotillomania | n/a |
|  | Excoriation Disorder | n/a |
| *Module N: Adjustment disorder* | Adjustment disorder | Current (past 6 months) |

*Note*. n/a = not available

**Appendix D. Additional items**

***LEC-5***

|  | **Event** | Happened to me | Witnessed it | Learned about it | Part of my job | Not Sure | Doesn’t Apply |
| --- | --- | --- | --- | --- | --- | --- | --- |
| 1. | Emotional abuse (like severely bullied, humiliated, yelled at, verbally threatened, punished in a unfair or cruel way) |  |  |  |  |  |  |
| 2. | Emotional neglect (like taking care of parents or other children in the family, parents were addicted to alcohol or drugs, being left to your own devices) |  |  |  |  |  |  |
| 3. | Physical neglect (like not getting enough food, or need to provide for your own food, left home alone during the day or at night under age of 12, not getting the medical care that was necessary) |  |  |  |  |  |  |

***PCL-5***

|  | | **With respect to your index trauma:**  **____________________** *(index trauma)* | | | | |  | **With respect to all your traumatic experiences excluding your index trauma** | | | | |
| --- | --- | --- | --- | --- | --- | --- | --- | --- | --- | --- | --- | --- |
| **In the past week, how much were you bothered by:** | | Not at all | A little bit | Moderately | Quite a bit | Extremely |  | Not at all | A little bit | Moderately | Quite a bit | Extremely |
| 1. | Feeling guilty? | 0 | 1 | 2 | 3 | 4 |  | 0 | 1 | 2 | 3 | 4 |
| 2. | Feeling ashamed? | 0 | 1 | 2 | 3 | 4 |  | 0 | 1 | 2 | 3 | 4 |
| 3. | Feeling angry? | 0 | 1 | 2 | 3 | 4 |  | 0 | 1 | 2 | 3 | 4 |
| 4. | Feeling disgusted? | 0 | 1 | 2 | 3 | 4 |  | 0 | 1 | 2 | 3 | 4 |
| 5. | Feeling sad? | 0 | 1 | 2 | 3 | 4 |  | 0 | 1 | 2 | 3 | 4 |
| 6. | Feeling afraid? | 0 | 1 | 2 | 3 | 4 |  | 0 | 1 | 2 | 3 | 4 |
|  | **In the past 7 days, to what extent:** |  |  |  |  |  |  |  |  |  |  |  |
| 7. | Did you feel happy? | 0 | 1 | 2 | 3 | 4 |  |  |  |  |  |  |

***TVIC***

How well could you see the scenes that have been described? *(read scale out loud)*

| Not at all | 0 100 | As vivid as if it was really happening |
| --- | --- | --- |

……

How difficult did you find it to visualize the different scenes? *(read scale out loud)*

| Not at all difficult | 0 100 | Extremely difficult |
| --- | --- | --- |

……

**Appendix E. Guilt and shame questionnaire**

This questionnaire is about different feelings that one might have. Please indicate for each feeling how frequent you experienced it during the last four weeks.

| **During the last four weeks** | never | 1 – 2 times | 3-4 times | More than once per week | daily |
| --- | --- | --- | --- | --- | --- |
| 1. I felt embarrassed | 0 | 1 | 2 | 3 | 4 |
| 1. I felt guilty | 0 | 1 | 2 | 3 | 4 |
| 1. I felt regret | 0 | 1 | 2 | 3 | 4 |
| 1. I felt disgusting | 0 | 1 | 2 | 3 | 4 |
| 1. I felt remorse | 0 | 1 | 2 | 3 | 4 |
| 1. I felt humiliated | 0 | 1 | 2 | 3 | 4 |
| 1. I felt ashamed | 0 | 1 | 2 | 3 | 4 |
| 1. I felt intense guilt | 0 | 1 | 2 | 3 | 4 |

**Appendix F. Memory task**

Think back to what happened in the previous session. Please describe as precisely as possible what are the most important things you remember happening during this session. Take maximum 10 minutes and write in the box below.

**Appendix G. Mediator items**

***Patient questionnaire***

Instruction: please answer each question by placing a vertical mark on the line on the place that reflects how you feel about the issue at the moment, that is now, just before the treatment session. After filling out the questions, please put them in a closed envelope and hand it over to the secretary (NOT to the therapist).

1. How motivated are you to do this treatment?

Not at all 0 100 Extremely

motivated motivated

1. How well do you feel that you and your therapist are working together?

Not at all 0 100 Extremely

working together well

1. How reluctant are you to participate in your treatment?

Not at all 0 100 Extremely

reluctant reluctant

1. How well do you remember what was addressed last session?

Not at all 0 100 Extremely well

1. How supported do you feel by your therapist?

Not at all 0 100 Extremely

supported supported

1. How well do you feel connected to your therapist?

Not at all 0 100 Completely connected connected

1. How difficult are you finding the therapy?

Not at all 0 100 Extremely

difficult difficult

1. Do you had enough time since the last session to process what you have learned/experienced?

Not at all 0 100 Completely

1. Since the last session, have there been changes in your life or other daily stressful experiences that interfere with your ability to do the therapy?

Not at all 0 100 Extreme

1. How optimistic are you that this therapy will be successful?

Not at all 0 100 Extremely

optimistic optimistic

***Therapist questionnaire***

Instruction: please answer each question by placing a vertical mark on the line on the place that reflects how you feel about the issue at the moment, that is now, just before the treatment session. The questions relate to this specific patient (‘s treatment).

1. How motivated are you to do this treatment?

Not at all 0 100 Extremely

motivated motivated

1. How well do you feel that you and your patient are working together?

Not at all 0 100 Extremely

working together well

1. How reluctant are you to deliver this treatment?

Not at all 0 100 Extremely

reluctant reluctant

1. How well do you remember what was addressed last session?

Not at all 0 100 Extremely well

1. How much do you feel you support your patient?

Not at all 0 100 Extremely

1. How well do you feel connected to your patient?

Not at all 0 100 Completely connected connected

1. How difficult are you finding the therapy?

Not at all 0 100 Extremely

difficult difficult

1. Do you think that your patient had enough time since the last session to process what (s)he has learned/experienced?

Not at all 0 100 Completely

1. How optimistic are you that this therapy will be successful?

Not at all 0 100 Extremely

optimistic optimistic

**Appendix H. Willingness to talk about traumatic experiences**

Please answer the question by placing a vertical mark on the line on the place that best describes how much you agree or disagree with each statement.

| 1. I don’t like to talk about my traumatic experiences. | | |
| --- | --- | --- |
| Totally disagree | 0 100 | Totally agree |
| 1. I would like to talk with someone about my traumatic experiences. | | |
| Totally disagree | 0 100 | Totally agree |

**Appendix I. Avoidance of shame and guilt**

Please answer the question by placing a vertical mark on the line on the place that best describes how you feel about this.

| 1. I avoid sharing issues that I feel ashamed about. | | |
| --- | --- | --- |
| Never | 0 100 | Always |
| 1. I avoid sharing issues that I feel guilty about. | | |
| Never | 0 100 | Always |

**Appendix J. Study board members**

**Roles and responsibilities**

The study is guided by the study board, which acts as the steering committee. The study board is composed of the principal investigator, co-principal investigator, junior investigators, and site investigators. The principal investigator is the overall lead researcher of the trial. Both the principal investigator and co-principal investigator are responsible for 1) the initial conception and design of the trial, 2) the overall conduct of the trial, 3) preparations of protocols, and 4) organizing study board meetings. The junior investigators are involved in the implementation of the data collection (Carlijn J. M. Wibbelink) and the coordination of the data collection (Sophie A. Rameckers), including keeping an overview of the progress, collecting records of all participants, and interacting with the local research assistants performing the assessments. The site investigators are responsible for the conduct of the trial at their mental healthcare center.

**Trial investigators**

*Principal investigator*

Arnoud Arntz

*Co-principal investigator*

Christopher W. Lee

*Junior investigators*

Sophie A. Rameckers

Carlijn J. M. Wibbelink

*Site investigators*

At the time of writing, the following trial investigators were involved:

Nathan Bachrach

Sarah K. Dominguez

Thomas Ehring

Saskia M. van Es

Eva Fassbinder

Sandra Köhne

Magda Mascini

Marie-Louise Meewisse

Simone Menninga

Nexhmedin Morina

Kathleen Thomaes

Carla J. Walton

Ingrid G. Wigard
